# The Interaction of CCDC104/BARTL1 with Arl3 and Implications for Ciliary Function

**DOI:** 10.1016/j.str.2015.08.016

**Published:** 2015-11-03

**Authors:** Mandy Lokaj, Stefanie K. Kösling, Carolin Koerner, Sven M. Lange, Sylvia E.C. van Beersum, Jeroen van Reeuwijk, Ronald Roepman, Nicola Horn, Marius Ueffing, Karsten Boldt, Alfred Wittinghofer

**Affiliations:** 1 Max-Planck-Institute of Molecular Physiology, Emeritus Group, Otto-Hahn-Straße 15, 44227 Dortmund, Germany; 2 Department of Human Genetics and Radboud Institute for Molecular Life Sciences, Radboud University Medical Center, Geert Grooteplein Zuid 10, 6525 GA Nijmegen, the Netherlands; 3 Medical Proteome Center, Institute for Ophthalmic Research, University of Tübingen, Nägelestrasse 5, 72074 Tübingen, Germany

## Abstract

Cilia are small antenna-like cellular protrusions critical for many developmental signaling pathways. The ciliary protein Arl3 has been shown to act as a specific release factor for myristoylated and farnesylated ciliary cargo molecules by binding to the effectors Unc119 and PDE6δ. Here we describe a newly identified Arl3 binding partner, CCDC104/CFAP36. Biochemical and structural analyses reveal that the protein contains a BART-like domain and is called BARTL1. It recognizes an LLxILxxL motif at the N-terminal amphipathic helix of Arl3, which is crucial for the interaction with the BART-like domain but also for the ciliary localization of Arl3 itself. These results seem to suggest a ciliary role of BARTL1, and possibly link it to the Arl3 transport network. We thus speculate on a regulatory mechanism whereby BARTL1 aids the presentation of active Arl3 to its GTPase-activating protein RP2 or hinders Arl3 membrane binding in the area of the transition zone.

## Highlights

• Identification of a BART-like protein, CCDC104/BARTL1, as Arl3 binding partner
• BARTL1 is a ciliary protein with transition zone localization
• Complex structure shows BARTL1 binding to conserved LLxILxxL N-term motif of Arl3
• N-terminal amphipathic helix of Arl3 is crucial for its ciliary localization

## Graphical Abstract

**Figure:**
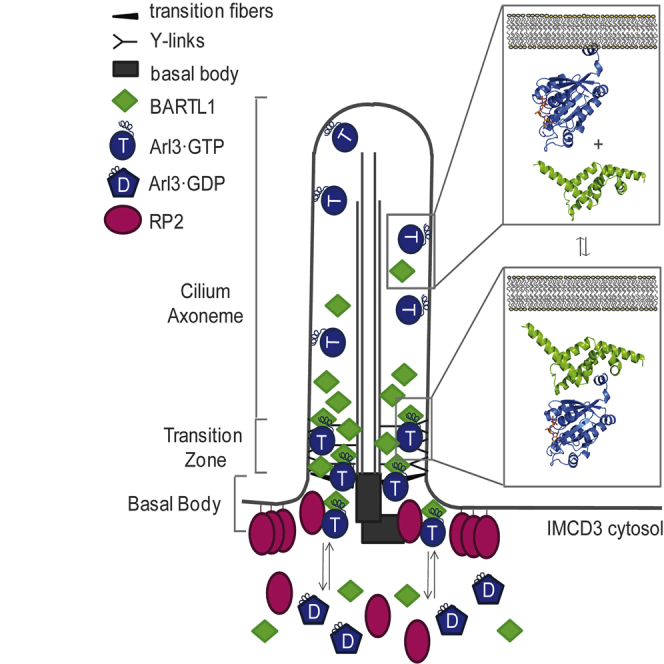

## Teaser

Lokaj et al. biochemically and structurally characterize BARTL1 as an Arl3 effector and speculate on its function in cilia.

## Introduction

Cilia are small, microtubule-based antennae-like protrusions of cells critical for the maintenance of cellular homeostasis and many developmental signaling pathways (Eggenschwiler and Anderson, 2007, Goetz and Anderson, 2010). Small G proteins of the Arl subfamily have been shown to be crucial to ciliogenesis and cilia maintenance. Joubert syndrome, Bardet-Biedl syndrome, and retinitis pigmentosa are so-called ciliopathies, arising from structural and/or functional defects of the G proteins Arl13B (Cantagrel et al., 2008, Thomas et al., 2015), Arl6 (Fan et al., 2004), and Arl3 (Schwahn et al., 1998, Veltel and Wittinghofer, 2009, Veltel et al., 2008a), respectively.

Arl2 and Arl3 (Arf-like) are guanosine triphosphate (GTP)-binding proteins of the Arf subfamily of the Ras superfamily. They switch between an inactive guanosine diphosphate (GDP)-bound form and an active GTP-bound form (Cox and Der, 2010, Vetter and Wittinghofer, 2001). This molecular switch is particularly striking for all (hitherto analyzed) members of the Arf subfamily, as it involves the reorganization of the β sheet, where two strands of the sheet move by two residues along the rest of the strands when going from the inactive GDP state to the active GTP state (Gillingham and Munro, 2007, Pasqualato et al., 2001, Pasqualato et al., 2002). This so-called interswitch toggle has been demonstrated by a number of three-dimensional structures to release the N-terminal (usually) amphipathic helix from its binding site on the G domain core, such that it is pointing into solution and/or is free to interact with membranes and/or other proteins (Cherfils and Zeghouf, 2013).

Arl2 and Arl3 are homologous proteins with approximately 52% sequence identity (68% similarity) and very similar structure. In addition, numerous effectors have been identified which interact with the GTP-bound form of both proteins. These are the delta subunit of the photoreceptor-specific phosphodiesterase 6 (PDE6δ) (Linari et al., 1999), HRG4/Unc119a (Kobayashi et al., 2003), its homolog Unc119b (Wright et al., 2011), and the Arl2-binding protein (BART/Arl2BP) (Sharer and Kahn, 1999, Veltel et al., 2008b, Zhang et al., 2009). The structure of the Arl2∙PDE6δ complex showed an Arf-type conformational change. The homology to the prenyl-binding protein RhoGDI (Hanzal-Bayer et al., 2002) led to the discovery that PDE6δ, also called PrBP, is a general prenyl-binding protein which seems to bind both farnesylated and geranylgeranylated proteins with unclear specificity (Chandra et al., 2012, Nancy et al., 2002, Zhang et al., 2004). Later it was shown that Arl2/3 and cargo binding are mutually exclusive and that Arl2/3 act as allosteric cargo-release factors by inducing a conformational change on PDE6δ (Ismail et al., 2011). HRG4/Unc119a has a sequence and structural homology to PDE6δ and was shown to bind myristoylated cargo such as transducin-α (Wright et al., 2011). Unc119a and Unc119b seem to be general myristoyl-binding proteins, and Arl2 and Arl3 can both act as cargo-release factors, although the conformational change leading to release of cargo is rather different from that of PDE6δ (Ismail et al., 2012). While the structure of the Arl2∙BART complex revealed a novel recognition motif of an effector (Zhang et al., 2009), where BART binds the Arl2 N-terminal helix apart from the switch region, the function of BART/Arl2BP remains to be determined.

Despite the homology in structure and biochemistry, Arl2 and Arl3 may have entirely different biological functions. It was shown very early that transfection of GTPase-negative versions (Q→L) of Arl2/3 and the knockdown by RNAi differentially affect microtubule-dependent processes (Tian et al., 2010, Zhou et al., 2006). Arl2 has been shown to bind to tubulin cofactor D, a protein necessary for folding and/or formation of the polymerization-competent α,β-tubulin dimer (Bhamidipati et al., 2000, Shern et al., 2003).

Arl3 has been identified as a ciliary protein in bioinformatics screens and localization studies (Avidor-Reiss et al., 2004). The generation of *Arl3*-deficient mice revealed that Arl3 is indeed involved in ciliary function affecting kidney and photoreceptor development (Schrick et al., 2006). In support of this, Arl3 has been shown to be involved in flagellum integrity in *Leishmania* (Cuvillier et al., 2000). In human photoreceptor cells Arl3 is localized in the connecting cilium, a ciliary compartment important for the transport of components between inner and outer segments of photoreceptor cells (Grayson et al., 2002). Arl3, but not Arl2, can release myristoylated ciliary target proteins from their complex with Unc119 (Wright et al., 2011), and we have shown that the particular conformation of the N-terminal helix of Arl3 is responsible for this differential effect (Ismail et al., 2012). Likewise, it has been shown that the prenylated ciliary cargo protein INPP5E is released from its complex with the shuttle factor PDE6δ by Arl3 but not Arl2 (Thomas et al., 2014). In addition, we have shown that RP2, a gene mutated in X-linked retinitis pigmentosa, is a highly active and specific GTPase-activating protein acting on Arl3 but not Arl2 (Veltel et al., 2008a). In support of the role of RP2 in ciliary trafficking, the RP2 knockout mouse shows severe defects in trafficking of prenylated and myristoylated proteins (Schwarz et al., 2012a, Schwarz et al., 2012b, Wright et al., 2011, Zhang et al., 2015).

In our search for interacting ciliary proteins, we identified CCDC104/CFAP36 as an Arl3-interacting protein with structural homology to the binder of Arl2 (BART). BART has been found to be an Arl2-interacting protein (Sharer and Kahn, 1999), which is mutated in autosomal-recessive retinitis pigmentosa (Davidson et al., 2013). Here we investigate the functional and structural properties of CCDC104/CFAP36 as a new ciliary protein and Arl3 effector. Because of its homology to BART, we have renamed it BARTL1.

## Results

### CCDC104/BARTL1 Contains an N-Terminal BART-like Domain

**Figure 1:**
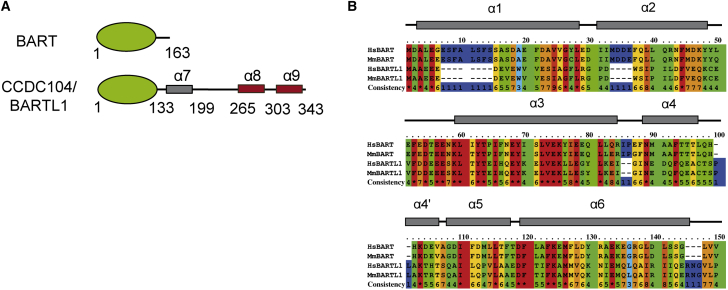
Domain Organization and Secondary Structure of BARTL1 (A) Domain organization of human BART and human BARTL1 with amino acid boundaries of the BART-like domain (green), random coiled coil (gray), and further α helices (red). (B) Alignment of residues 1–133, comprising the BART-like domain, from *Homo sapiens* (Hs) and *Mus musculus* (Mm) BART and BARTL1. Dependent on their degree of conservation, residues are colored from red (highly conserved) to blue (non-conserved). The α helices of the BART-like domain are indicated above.

In a search for ciliary regulators (guanine nucleotide exchange factors [GEFs] and GTPase-activating proteins) for Arl3, we carried out tandem-affinity purifications (TAPs) from HEK293T cells that were transfected with constructs coding for the fast cycling mutant Arl3^D129N^ containing a C-terminal Strep-flag tag. Such a mutant is expected to associate with GEFs and effectors, as we have shown previously in the identification of plant-specific RopGEFs (Berken et al., 2005). We repeatedly identified peptides of CCDC104 by mass spectrometry analysis of TAP eluates (Table S1). Although CCDC104 was previously identified in a TAP using constitutively active Arl3^Q71L^ (Wright et al., 2011), we speculated, based on our findings, that CCDC104 might be a GEF for Arl3. In assessing this role, however, CCDC104 showed no GEF activity toward Arl3 (Figure S1). Bioinformatics analysis of the domain structure of CCDC104 showed the presence of an N-terminal BART-like domain followed by an extended C terminus comprising a coiled coil (α7) and two further α helices (α8 and α9) (Figure 1A). The presence and similarity to BART led us to rename CCDC104 to BARTL1 (BART-like protein 1). Despite low amino acid sequence conservation between the BART-like domain of BARTL1 and BART, with only 21.4% identity and 41.4% similarity over 133 amino acids, the secondary structure prediction shows a conserved all-helical domain consisting of six α helices (Figure 1B). Thus, considering the five known common effectors of Arl2/3, we can group these into two types, where BARTL1 and BART form one group while PDE6δ, HRG4, and Unc119b constitute the second. The latter three, despite low primary sequence conservation, have an identical immunoglobulin β-sandwich fold. They form a group of guanine nucleotide dissociation inhibitor-like solubilizing factors, which are regulated by Arl2 and Arl3 small G proteins (Chandra et al., 2012, Hanzal-Bayer et al., 2002, Ismail et al., 2011, Ismail et al., 2012). The former two can also be grouped together based on their identical all-helical fold, although the molecular functions of BART and BARTL1 are presently unknown and BARTL1 is the focus of the present study.

### Arl3 and BARTL1 Localize to Cilia

**Figure 2:**
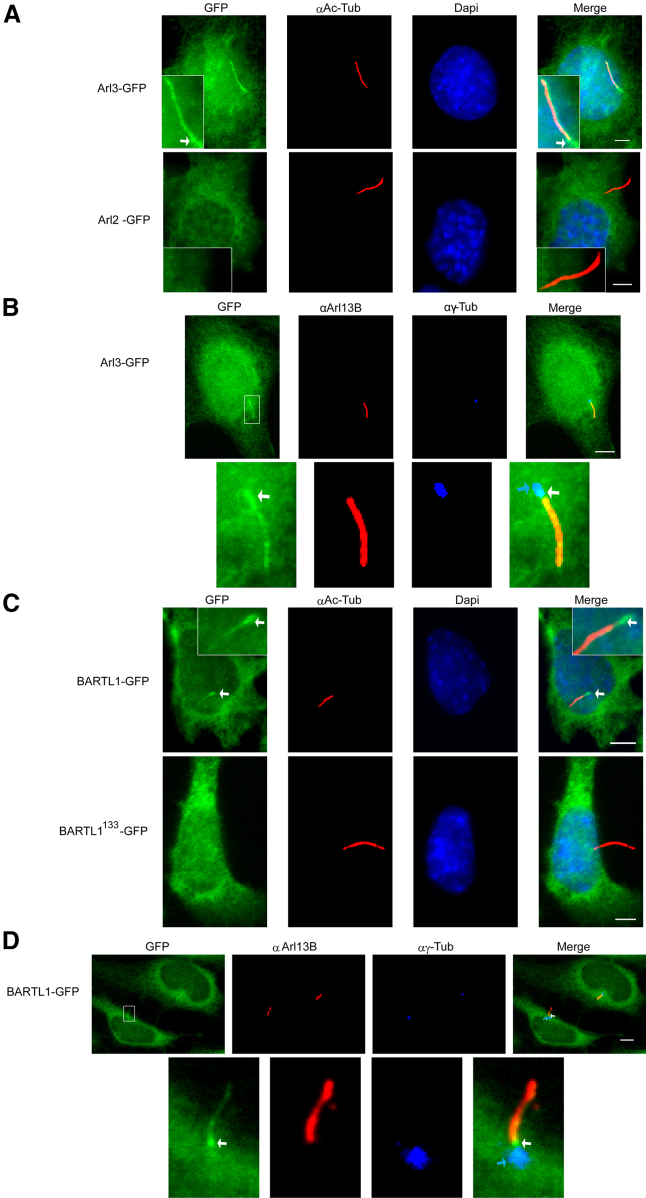
Localization of Arl2, Arl3, and BARTL1 in IMCD3 Cells with Induced Cilia (A) Stably expressed, C-terminally GFP-tagged full-length mouse Arl3 or Arl2 in IMCD3 Flp-In cells were serum-starved and fixed. Apart from GFP labeling (shown in all the following figures, as indicated), the cells were immunostained for acetylated α-tubulin (AcTub) and the nucleus (DAPI). Boxed areas show enlargement of cilia. White arrows point to the base of the cilium. (B) IMCD3 Flp-In cells stably expressing Arl3-GFP were stained for γ-tubulin (αγ-Tub; blue) and Arl13B (αArl13B; red). Indicated are basal body (blue arrow) and the GFP signal between basal body and Arl13B signal (white arrow). The boxed area in the upper row (left panel) is enlarged in the lower row. (C) IMCD3 Flp-In cells stably expressing C-terminally tagged human BARTL1-GFP and mouse BARTL1^133^-GFP were serum-starved, fixed, and immunostained for acetylated α-tubulin (AcTub) and the nucleus (DAPI). Boxed areas show enlargement of cilia. (D) IMCD3 Flp-In cells stably expressing BARTL1-GFP were stained for γ-tubulin (αγ-Tub; blue) and Arl13B (αArl13B; red). Indicated are basal body (blue arrow) and the GFP signal between basal body and Arl13B signal (white arrow). The boxed area in the upper row (left panel) is enlarged in the lower row. Scale bars represent 5 μm.

The cellular localizations of Arl3 and Arl2 were analyzed in mouse inner medullary collecting duct (IMCD3) cells. In agreement with the literature (Zhou et al., 2006), we confirm the ciliary localization for Arl3 along the length of the cilium, visualized by staining against acetylated α-tubulin of the cilia axoneme (Figure 2A), in addition to the rest of the cell, in IMCD3 Flp-In cell lines stably expressing Arl3 C-terminally fused to GFP. Examination of Arl3 staining by a different fixation method combined with staining of the cilia axoneme for Arl13B, which is a protein exclusively localizing to the cilia axoneme, and for γ-tubulin, which is a marker for the basal body, shows that Arl3 is also enriched at the basal body and the transition zone additional to the length of the cilium (Figure 2B). In contrast, a corresponding Arl2 construct was excluded from the cilium and could only be found in the cytoplasm (Figure 2A). This is further supported by reports that only Arl3 and not Arl2 is found in the ciliary proteome (Avidor-Reiss et al., 2004, Efimenko et al., 2005, Pazour et al., 2005). To examine a potential role of BARTL1 in cilia, we further generated cell lines stably expressing a C-terminal fusion to GFP. Following induction of cilia by serum starvation, native BARTL1 could be detected in cilia only partly, co-localizing with the ciliary marker acetylated α-tubulin (Figure 2C). It appears that BARTL1 is enriched at the base of the cilium (close to the basal body) (Figure 2C, white arrow). A closer investigation of the staining by a different fixation method combined with staining for γ-tubulin reveals that the enrichment of BARTL1 (Figure 2D, white arrow) appears distal to the basal body (Figure 2D, blue arrow), in the transition zone. Not surprisingly BARTL1 and Arl3 can be shown to co-localize, as discussed below (Figure S5).

Interestingly, the BART-like domain of BARTL1 is not sufficient to promote its ciliary localization, as the construct BARTL1^133^ is not found in cilia (Figure 2C). Hence, the C terminus of BARTL1 mediates and/or supports the localization to cilia by an as yet unknown mechanism. Whereas BART was reported to be localized at the basal body in photoreceptor cells (Davidson et al., 2013) and might be specifically expressed in photoreceptor cells, its localization in ciliated IMCD3 cells is variable and rarely in the cilium (data not shown). Moreover, BART has been reported to enter mitochondria and bind the adenine nucleotide transporter (Sharer et al., 2002). Based on our findings that BARTL1 and Arl3 are ciliary proteins, we postulate a role for BARTL1 in regulating the ciliary localization or function of Arl3, or vice versa.

### BART-like Domain of BARTL1 Is Sufficient to Promote Interaction with Arl3

**Figure 3:**
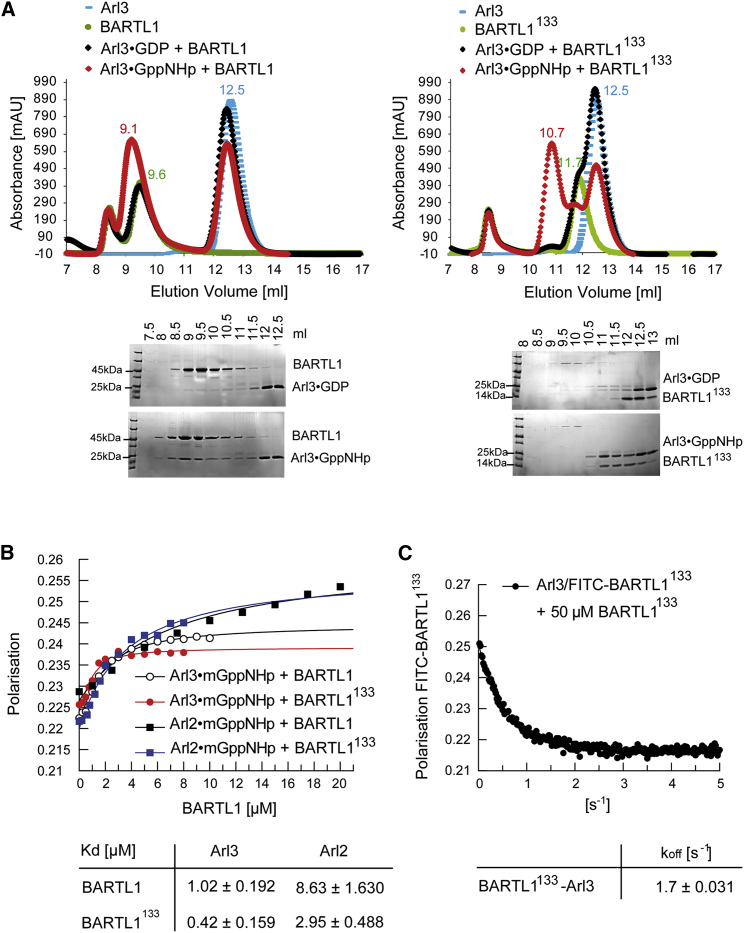
Biochemical Characterization of the BARTL1∙Arl Interaction (A) Analytical size-exclusion chromatography (Superdex75 10/300Gl). Elution profiles of BARTL1 full-length (green, left) or BARTL1^133^ (green, right) alone or mixed with Arl3 full-length bound to GDP (black) or GppNHp (red) as indicated. Elution profile of Arl3 alone is shown in blue. Elution fractions were analyzed by SDS-PAGE and Coomassie staining as shown below the graphs. (B) Determination of dissociation constants (*K*_D_) by fluorescence polarization measurements at 20°C in buffer M. 1 μM Arl3 or Arl2 full-length bound to mant-GppNHp were titrated with increasing amounts of BARTL1 full-length or BARTL1^133^. Fitting to a quadratic equation gives the dissociation constants (and standard deviations) shown in the table below the graph. (C) Stopped-flow fluorescence polarization at 20°C in buffer M. A preformed complex of 1 μM FITC-BARTL1^133^ with 2 μM Arl3-GppNHp was shot together with a 50-fold excess of unlabeled BARTL1^133^. The curve was fitted to a single exponential to determine the *k*_off_ rate (and SD), which is given below the graph.

We further investigated the interaction of BARTL1 with Arl3 rather than Arl2, since the former is the focus of our studies on ciliary trafficking. Based on the elution profile of an analytical gel filtration column, we demonstrated that BARTL1 forms a tight complex with Arl3, which is dependent on its nucleotide state (Figure 3A, left panel). Arl3 in its active GppNHp-bound but not in its inactive GDP-bound state forms a complex with BARTL1, which elutes at 9.1 ml compared with 9.6 ml for BARTL1 alone. To find out whether the full-length BARTL1 is necessary for the interaction with Arl3, we tested whether BARTL1^133^, comprising only the BART-like domain, is sufficient for binding to Arl3. Just as for full-length BARTL1, only Arl3 in its active GppNHp-bound state forms a complex (elution volume 10.7 ml of complex versus 11.7 ml of BARTL1^133^ alone) with the truncated protein (Figure 3A, right panel).

For a more quantitative analysis, dissociation constants (*K*_D_) were determined by titrating 1 μM Arl3 bound to mant-GppNHp with increasing amounts of effector and measuring fluorescence polarization. Complex formation increases the fluorescence polarization signal and shows that Arl3 binds to BARTL1 or BARTL1^133^ with *K*_D_ of 1 or 0.43 μM, respectively (Figure 3B). Arl2 displays a 10-fold lower affinity to BARTL1 or BARTL1^133^. Since affinity is usually dictated by the dissociation rate, we determined the dissociation rate constants *k*_off_ of Arl3 from BARTL1^133^ by stopped-flow fluorescence polarization measurements employing fluorescein isothiocyanate (FITC)-labeled BARTL1. The *k*_off_ for the complex is 1.7 s^−1^, which would give an association rate constant of 4 × 10^6^ M^−1^ s^−1^ for the interaction between Arl3 and BARTL1, within the normal range for a protein-protein interaction (Wohlgemuth et al., 2005).

### Structure of the Complex between BARTL1 and Arl3

**Figure 4:**
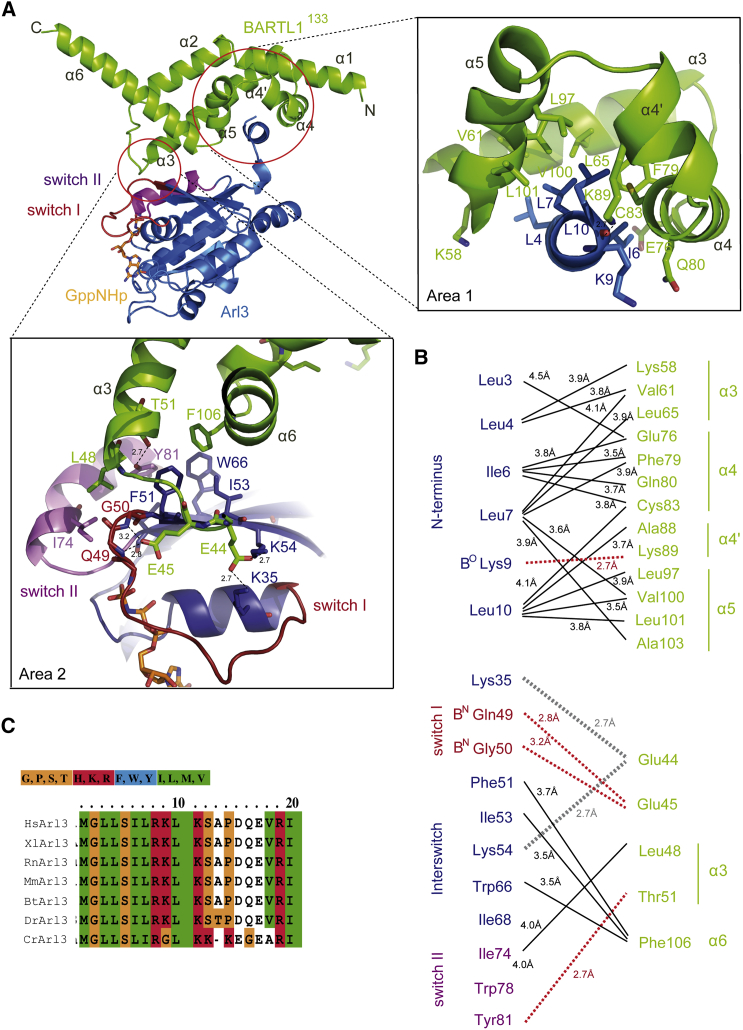
Structure of the Arl3∙GppNHp∙BARTL1^133^ Complex (A) Overview (left) and zoom-in of the interaction area 1 (right), with N-terminal helix of Arl3 (blue) buried in a hydrophobic groove of BARTL1^133^ (green). Zoom-in of interaction area 2 (below) shows BARTL1^133^ contacting switches I (red) and II (purple) of Arl3. α Helices of BARTL1^133^are numbered. (B) Schematic overview of residues from BARTL1^133^ (green) and Arl3 (blue) involved in the interaction: hydrophobic van der Waals interactions (solid black lines) involving the side chains of the residues indicated, H bonds (red dotted lines), and salt bridges (gray dotted lines). H bonds to backbone oxygen or nitrogen of residues are indicated by B^O^ or B^N^, respectively. Distances are indicated in angstroms. (C) Alignment of N terminus of Arl3 from different organisms, *Homo sapiens* (Hs), *Mus musculus* (Mm), *Rattus norvegicus* (Rn), *Xenopus laevis* (Xl), *Bos taurus* (Bt), *Danio rerio* (Dr), *Caenorhabditis elegans* (Ce), and *Chlamydomonas rheinhardtii* (Cr), shows that the N-terminal hydrophobic LLxILxxL motif is highly conserved in Arl3. Amino acids are colored according to the residue identity. Hydrophobic residues are shown in green.

The complex of BARTL1^133^ with full-length Arl3 bound to the non-hydrolyzable GTP analog GppNHp crystallized in space group P2_1_2_1_2_1_, and diffracted to 2.2 Å resolution (Table 1; PDB: 4ZI2). The asymmetric unit contained two Arl3∙GppNHp and two BARTL1^133^ molecules (Figure S2A). BARTL1^133^ displays the same all-helical fold as seen in BART (Zhang et al., 2009) (Figure 4A). The nomenclature of the α helices was adjusted according to the BART structure (PDB: 3DOE). Part of the BART structure in the Arl2∙GTP∙BART complex (PDB: 3DOE) (Zhang et al., 2009) was not visible in the electron density. However, in BARTL1^133^ it was visible and termed helix α4′, which is situated at a right angle to α4 (Figure 4A). The side chain of residue Lys89^B^ (superscript B stands for BARTL1, A for Arl3) from α4′ forms a hydrogen bond with the backbone oxygen of Lys9^A^ (Figure 4A, right zoom Area1), which might explain why the α4′ helix of BARTL1^133^ as well as the N-terminal helix of Arl3 are less flexible and could thus be traced in the electron density.

**Figure 5:**
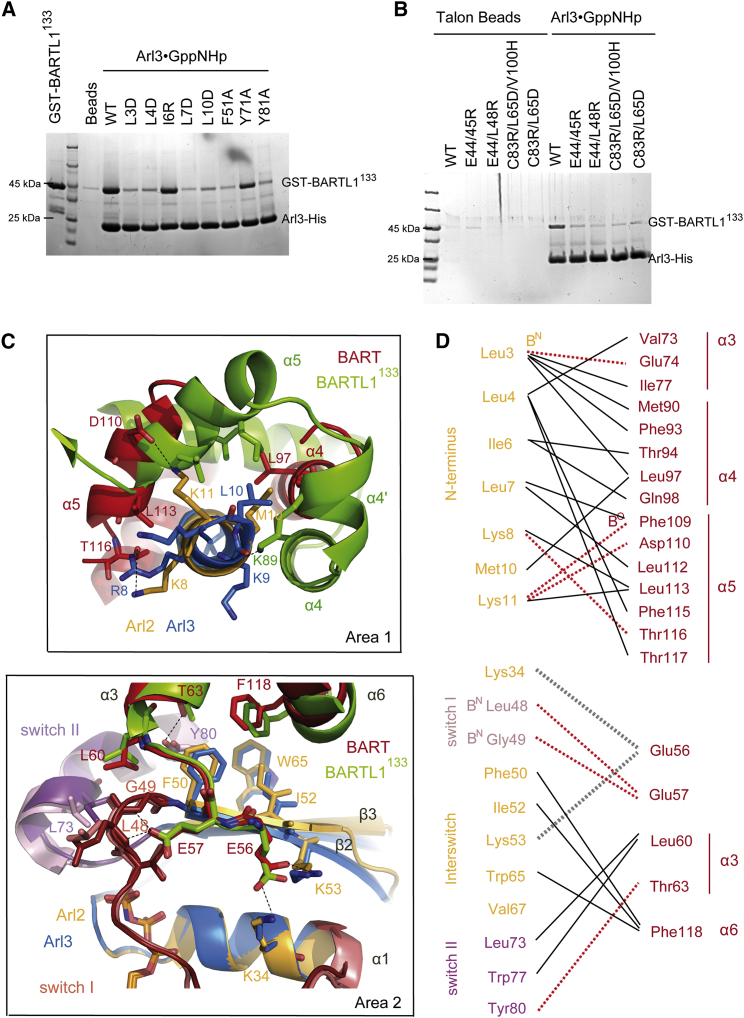
Biochemical Characterization of the Interface of the Arl3∙GppNHp∙BARTL1^133^ Complex and Comparison with Arl2∙GTP∙BART (A) Pull-down of GST-BARTL1^133^ wild-type by either wild-type or mutant Arl3-His bound to Talon beads. (B) Pull-down of GST-BARTL1^133^ wild-type or mutants by Arl3-His bound to Talon beads. (C) Interaction area 1 (upper) as in Figure 3A from the BARTL1^133^ (green) or BART (red) complexes obtained by superimposing the N-terminal helices of Arl3 (blue) with Arl2 (orange), respectively; interaction area 2 (below) as in Figure 3A, obtained by superimposition of Arl2 and Arl3, shows contact of switches I (red) and II (purple) of Arl3 or Arl2, with BARTL1^133^ or BART, respectively. (D) Schematic overview of residues from BART (red) and Arl2 (orange) involved in the interaction interface as described in Figure 4B.

To distinguish crystal packing contacts from the correct Arl3∙BARTL1 interface, we compared it with the structure of Arl3∙GppNHp∙BARTL1^133^ in space group P2_1_, which was solved at 2.0 Å resolution (PDB: 4ZI3; Table 1 and Figure S2B). Comparison of these structures with that of Arl2∙GTP∙BART (PDB: 3DOE; Figure S2C) (Zhang et al., 2009) led us to postulate two areas contributing to the Arl3∙BARTL1 interface (Figure 4A). As expected for an effector of small G proteins, BARTL1 is in contact with the switch regions of Arl3 (area 2). In addition, BARTL1 completely buries the N-terminal helix of Arl3 (area 1). This unconventional binding mode sets it apart from effectors such as PDE6δ or Unc119 and from many other effectors of the Ras superfamily proteins (Wittinghofer and Vetter, 2010). The hydrophobic side of the N-terminal amphipathic helix of Arl3 is buried in a hydrophobic groove (Figures 4 and S2D) formed by helices α3, α4, α4′, and α5 of BARTL1. Leu3^A^, Leu4^A^, Ile6^A^, Leu7^A^, and Leu10^A^ are submerged in a hydrophobic patch made up by Lys58^B^, Val61^B^, Leu65^B^, and Leu69^B^ on α3, Phe79^B^ and Cys83^B^ on α4, Ala88^B^ on α4′, and Leu97^B^, Val100^B^, and Leu101^B^ on α5 (BARTL1 [B] and Arl3 [A]; Figures 4A area 1, and 4B). Alignment of the Arl3 N-terminal sequence of different species shows a conserved LLxILxxL motif (Figure 4C). A similar motif is found in the Arl2∙GTP∙BART complex. To define the contribution of these residues to the interaction, conserved residues in the _3_LLxILxxL_10_ motif of the Arl3 N-terminal helix were mutated, and the mutated proteins analyzed in a pull-down assay. Binding to GST-BARTL1^133^ was disrupted for the mutants Arl3^L3D^, Arl3^L4D^, Arl3^L7D^, and Arl^L10D^. Surprisingly, even though Ile6 is also pointing into the hydrophobic core of the interface, the Arl3^I6R^ mutation does not change the affinity (Figure 5A).

The second interface area is formed by switch I, switch II, and residues of the interswitch toggle of Arl3, and on the BARTL1^133^ side by the loop connecting α2 and α3 as well as parts of the α3 and α6 helices (Figure 4A, area 2). Hydrophobic interactions involving Phe51^A^ and Ile53^A^ of β2, Trp66^A^ of β3, Ile74^A^, Tyr81^A^ in switch II, and Phe106^B^ of α6, and Leu48^B^ and Thr51^B^ of α3 seem to be important. There are polar interactions between Thr51^B^ and Tyr81^A^, switch I main-chain nitrogens of Gln49^A^ and Gly50^A^ with Glu45^B^ and Glu44^B^ of the α2-α3 loop with Lys45^A^ of β2 in the interswitch toggle and Lys35^A^ in the α1 helix (Figures 4A and 4B). Mutations of F51A and Y81A in Arl3 in area 2 weaken the interactions with GST-BARTL1^133^ in a pull-down assay while Y71A seems to have no effect (Figure 5A). Introduction of single-residue mutations on the side of BARTL1^133^ were not sufficient to disturb the interaction, so double or triple mutations had to be introduced. The simultaneous mutation of Cys83, Leu65, and Val100 in the hydrophobic groove on the surface of BARTL1 weakens the interaction with Arl3. The loss of the polar interactions by the double mutant BARTL1^133 E44/45R^ also disrupts the interaction with Arl3 (Figure 5B).

### Arl3∙BARTL1 Complex Compared with Arl2∙BART

BARTL1 complexing with Arl3 displays similar recognition motifs as seen in the BART∙Arl2 crystal structure. Arl3 and Arl2 of both structures overlay with a root-mean-square deviation (rmsd) of 0.788 Ǻ^2^ over 165 residues (Figure S3, left), whereas BARTL1 and BART superimpose with an rmsd of 3.521 Ǻ^2^ over 94 residues (Figure S3, right). Focusing on the superimposition of Arl2/3, the core G domains align nearly perfectly, with the main differences in the conformation of the N and C termini and similar relative locations of BARTL1 and BART. Whereas fewer residues of the N and C terminus of BART are visible and the region between α4 and α5 helices is not resolved, these parts of BARTL1 can be traced (Figure S3 and Figure 5C, upper), partly due to the interaction between Lys89 side chain of BARTL1 from α4′ with the backbone oxygen of Lys9 of Arl3 (Figure 4A, see above). In contrast, the N-terminal helix of Arl2 seems to be anchored by an H bond of Glu74^BART^ with the backbone nitrogen of Leu3^Arl2^, an interaction not found in the Arl3∙BARTL1 complex. Further major differences in interaction area 1 are the polar interactions of Lys11^Arl2^ with Asp110^BART^, and Lys8^Arl2^ with Thr116^BART^, while Lys11 and Arg8 of Arl3 are not involved in any interactions (Figures 5C, upper and 5D). While in the Arl3∙BARTL1 structure more hydrophobic contacts are formed by Leu10, Leu7, and Ile6 of Arl3, in the Arl2∙BART structure Leu3 and Leu4 of Arl2 are involved in more hydrophobic interactions. Hence, Leu10 is more important in Arl3 and constitutes a conserved LLxILxxL motif while in Arl2 a conserved LLxIL motif is present, as was found by Zhang et al. (2009). The contact area 2 between the switch regions of Arl2/3 and BART/BARTL1 are nearly identical, as summarized in Figure 5D (lower).

### N-Terminal Helix of Arl3 Is Crucial for Interaction with BARTL1 and Essential for Its Ciliary Localization

**Figure 6:**
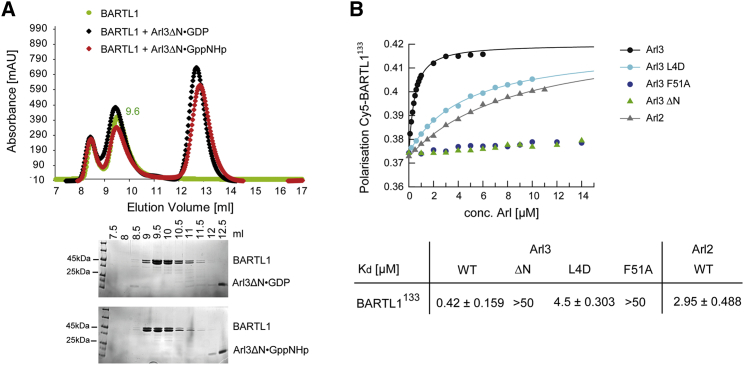
N-Terminal Helix of Arl3 is Crucial for the Interaction with BARTL1 (A) Analytical size-exclusion chromatography (Superdex75 10/300Gl) of BARTL1 alone (green) or mixed with Arl3ΔN bound to GDP (black) or GppNHp (red) as indicated. Elution fractions were analyzed by SDS-PAGE and Coomassie staining as shown below the graph. (B) Determination of dissociation constants (*K*_D_) by fluorescence polarization measurements at 20°C in buffer M. 200 nM Cy5-BARTL1^133^ was titrated with increasing amounts of Arl3^WT^, Arl3^L4D^, Arl3^F51A^, Arl3^ΔN^, and Arl2^WT^. Fitting to a quadratic equation gives the dissociation constants (and SDs) shown in the table below the graph.

Based on the presence of a conserved N-terminal sequence in Arl3 and mutational analysis mentioned above, we hypothesized that the N-terminal helix is crucial for the interaction of Arl3 with BARTL1. Deletion of the N terminus leads to complete loss of complex formation. The elution profile of an analytical gel filtration shows no complex formation of BARTL1^133^ with Arl3ΔN in its active GppNHp-bound state (Figure 6A). To more quantitatively describe the effect of the mutation, we carried out fluorescence polarization measurements using Cy5-labeled BARTL1^133^. Our results support the notion that the absence of the Arl3 N terminus leads to a *K*_D_ higher than 50 μM, representing a more than 100-fold loss in affinity (Figure 6B). The mutation of the N-terminal residue Leu4 in Arl3 reduces affinity by 10-fold (Figure 6B), indicating that a single mutation within the hydrophobic motif _3_LLxILxxL_10_ is not enough to mimic the deletion of the whole Arl3 N terminus. Since the mutant protein Arl3^F51A^ shows a similar drastic, more than 100-fold loss of affinity, we can conclude that both contact areas make significant contributions to the affinity of the interaction.

**Figure 7:**
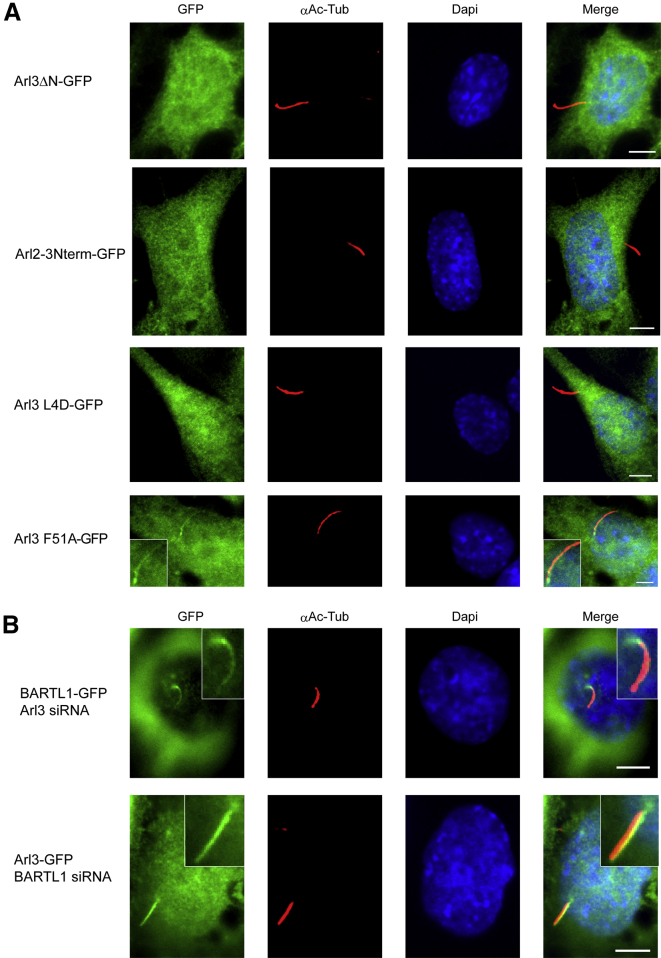
Localization of Arl3 Mutants in IMCD3 Cells and Knockdown of Arl3 and BARTL1, Using the Presentation Scheme as Explained in Figure 2 (A) Stably expressed, C-terminally GFP-tagged mouse Arl3^ΔN^, Arl2^3Nterm^, Arl3^L4D^, and Arl3^F51A^ in IMCD3 Flp-In cells were immunostained for acetylated α-tubulin (AcTub) and the nucleus (DAPI) as indicated. (B) Transient knockdown of BARTL1 in IMCD3 Flp-In cells stably expressing Arl3-GFP (upper panels) and knockdown of Arl3 in cells stably expressing BARTL1-GFP (lower panels). The efficiency of knockdown was analyzed by western blot of cell lysates, and is shown in Figure S4.

To investigate whether the ciliary localization of Arl3 and BARTL1 is dependent on their interaction, we generated various stable IMCD3 Flp-In cell lines. Deletion of the Arl3 N-terminal helix leads to a complete loss of the ciliary localization of Arl3. A C-terminal GFP fusion construct of Arl3ΔN compared with full-length Arl3 shows no GFP signal in cilia and lacks complete co-localization with the ciliary marker acetylated α-tubulin (Figure 7A). Hence, the N terminus of Arl3 seems to be part of or the complete ciliary localization signal. This result is surprising and raises the question why Arl2, despite 52% identity to Arl3 and only minor differences in its N-terminal sequence, is not a ciliary protein. We generated a chimera of the Arl2 G domain fused to the N-terminal 17 amino acids of Arl3 (Arl2-3Nterm), which failed to localize to cilia (Figure 7A). We concluded that the Arl3 N terminus is not sufficient to mediate localization to cilia and that the full context of the Arl3 protein is required instead (Ismail et al., 2012). This seems to indicate that a specific retention signal is required for the ciliary localization of Arl3.

We therefore hypothesized that an effector binding to the N terminus of Arl3, such as BARTL1, is either crucial to mediate the transport of Arl3 into cilia or is important to retain Arl3 within cilia, an assumption that is supported by the co-localization of the two proteins. We thus generated cell lines stably expressing GFP-tagged Arl3^L4D^ and Arl3^F51A^ mutants, which have defects in binding to BARTL1 as demonstrated above. Arl3^L4D^ completely failed to localize to cilia (Figure 7A). Notably, the cilia length was also reduced in cell lines expressing Arl3^L4D^-GFP compared with Arl3^WT^-GFP (Figure S4A). Arl3^L4D^ decreases affinity to BARTL1 by 10-fold, so this effect could potentially be attributed to a weakened interaction. However, in contrast to our expectations, the mutant Arl3^F51A^ with a drastically reduced affinity to BARTL1 shows no defects in localization or cilia length (Figures 7A and S4A). We can thus conclude that the interaction with BARTL1 is not responsible for ciliary localization. We may also conclude, however, that the L4D mutation does disrupt the binding of Arl3 to membranes, which is heavily dependent on the N-terminal amphipathic helix (our unpublished data). For further analysis, we performed knockdown experiments. A knockdown of Arl3 had no effect on the localization of a C-terminal GFP fusion construct of BARTL1 (Figures 7B and S4B). Hence, it can be concluded that Arl3 is not regulating the localization of BARTL1. A knockdown of BARTL1 in Arl3 stable cell lines also showed no effect (Figures 7B and S4B), although it cannot be excluded that small interfering RNA (siRNA) knockdown did not result in a complete abolition of the relevant protein levels and therefore led to no observable cellular phenotype (Figure S4B).

## Discussion

Here, we demonstrate by X-ray structure determination that BARTL1 binds Arl3∙GppNHp in a similar fashion to BART complexing Arl2∙GTP (Zhang et al., 2009). It was previously shown that BART is an Arl2/3 effector (Sharer and Kahn, 1999, Veltel et al., 2008b, Zhang et al., 2009), as we demonstrate here for BARTL1. Therefore, both BART and BARTL1 form a group of Arl2/3 effectors displaying an all-helical BART domain with an unconventional recognition mode involving the binding of the N-terminal helix of Arl2/3 apart from the switches.

This binding mode is clearly different from the second group of Arl2/3 effectors formed by PDE6δ, HRG4, Unc119a, and Unc119b. These effectors display an immunoglobulin β-sandwich fold and bind to the switch regions of Arl2/3, thereby continuing the central β sheet of the Arl G protein. The structure of Arl3∙Unc119a shows that the N-terminal helix of Arl3 is not contacting the effector but is important for the release of myristoylated cargo from Unc119a (Ismail et al., 2012). Biochemically we show that the N terminus of Arl2 does not affect cargo release.

Having shown that BARTL1 is a bona fide effector that binds to the GTP-bound form of Arl3 (and Arl2), we set out to speculate on the role of this interaction. We show here that both Arl3 and BARTL1 seem to be ciliary proteins with a partly overlapping localization. While Arl3 is co-staining with acetylated α-tubulin over the entire length of the cilia axoneme and seems concentrated at the transition zone, BARTL1 co-localizes with Arl3 distal to the basal body, corresponding to the transition zone, localized between basal body and cilia axoneme, as shown by co-staining with γ-tubulin as a basal body marker. Staining of endogenous Arl3 in a stable cell line expressing BARTL1-GFP confirms that both proteins are present in the cilia axoneme and around the transition zone (Figure S5). In addition, we have shown here by knockdown experiments that this localization is not dependent on the presence of either of the two proteins.

**Figure 8:**
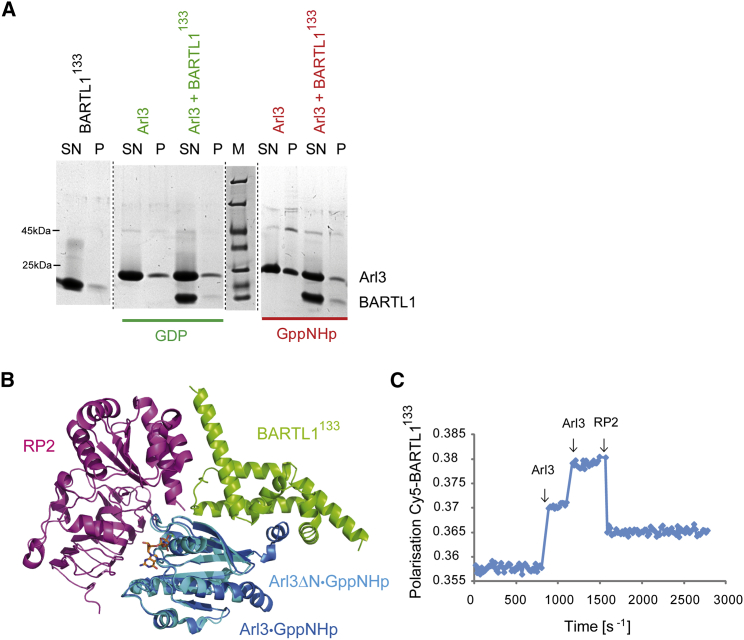
Investigation into Possible Function of BARTL1 (A) Liposome sedimentation assay. 2.8 mM of 200-μm liposomes of DOPC/DOPG/DPPC/DPPG/cholesterol composition were incubated with 20 μM Arl3 bound to GDP or GppNHp in the presence of 40 μM BARTL1^133^. Aliquots of the supernatant (SN) and pellet (P) compared with the marker (M) following sedimentation were analyzed by SDS-PAGE. (B) Overlay of Arl3∙GppNHp∙RP2 (PDB: 3BH6) with Arl3∙GppNHp∙BARTL1^133^ (PDB: 4ZI2). (C) Fluorescence polarization measurements at 20°C in buffer M: 1 μM Cy5-BARTL1^133^ was titrated twice with 1 μM Arl3∙GppNHp, followed by addition of 10 μM RP2 (as indicated by arrows).

We would like to propose two possible, though not necessarily mutually exclusive, roles for the Arl3-BARTL1 interaction. It has been shown by us and others that the GTP-bound form of Arl3 releases farnesylated and myristoylated ciliary cargo from the transport factors PDE6δ and Unc119a/b. Since this is required for cargo to be transported into cilia, Arl3 is most likely localized as Arl3∙GTP inside cilia. The exclusive localization of active Arl3 inside cilia is guaranteed by the Arl3-specific GTPase-activating protein RP2, which we find enriched around the basal body in IMCD3 cells (Figure S6A). We thus propose that the role of BARTL1 might be to prevent or reduce membrane interaction of Arl3∙GTP and mediate the GTP hydrolysis of Arl3∙GTP by RP2. We have shown that the nucleotide state and the presence of the N terminus are important for the membrane interaction of Arl3 (K.W., unpublished data). This is supported by a liposome sedimentation assay, whereby more Arl3 in its active GppNHp-bound state is precipitated than in its inactive GDP-bound state, representing the fraction bound to liposomes (Figure 8A). Addition of BARTL1^133^ to Arl3 reduces the association of Arl3-GppNHp with liposomes.

Furthermore, superimposing the Arl3∙BARTL1 (PDB: 4ZI2) structure with that of the Arl3ΔN∙RP2 complex (PDB: 3BH6; Figure 8B) (Veltel et al., 2008a) shows that a triple complex between the three components can in principle be formed. Such a complex would, however, be very transient, since the addition of RP2 to an Arl3∙GppNHp∙BARTL1^133^ complex leads to dissociation, as shown by fluorescence polarization using Cy5-labeled BARTL1^133^ (Figure 8C). This experiment suggests a displacement of Arl3-GppNHp from Cy5-BARTL1^133^ and formation of an Arl3∙GppNHp∙RP2 complex. An interaction between Cy5-BARTL1^133^ and RP2 could not be observed (data not shown) although we cannot exclude that the C terminus of BARTL1 might play a role in this interaction. Addition of Arl3 to full-length Cy5-BARTL1 showed no signal change, and therefore could not be used to test for triple complex formation (data not shown). Although BARTL1 does not influence either the intrinsic or the RP2-stimulated GTP hydrolysis of Arl3 (Figure S6B), the localization of BARTL1 on top of the RP2 domain might still mediate the exit of Arl3 as an Arl3∙GTP complex from the cilium through the transition zone toward the basal body, followed by GTP hydrolysis mediated by RP2. Such a scenario might also be responsible for creating an energetic driving force for the entry of cargo into cilia, just as Ran∙GTP hydrolysis is the driving force for nucleocytoplasmic transport across the nuclear pore.

## Experimental Procedures

See Supplemental Experimental Proceduresfor plasmids and protein purification, Cy5 and FITC labeling of BARTL1, tandem affinity purification, mass spectrometry and liposome sedimentation assay.

### Crystallization

Native full-length Arl3 was purified and exchanged as previously described to be completely loaded with GppNHp (Veltel et al., 2006, Veltel et al., 2008b). Arl3∙GppNHp was mixed with BARTL1^133^ in a molar ratio of 1.3 to 1 at 16.7 mg/ml. The sitting-drop/vapor diffusion method was used, and initial conditions were established in EasyXtal CORE II Suite (1 M LiCl, 0.1 M MES [pH 6.0], 30% polyethylene glycol [PEG] 6000) and EasyXtal PEG II Suite (1 M LiCl, 0.1 M Tris [pH 8.5], 20% PEG 4000) from Qiagen. Crystals appeared after 1–3 days and were flash-frozen after 3 days from a 96-well screen in cryosolution containing the same constituents as the crystallizing condition supplemented with 20% glycerol. Crystals from the CORE II Suite were of space group P2_1_2_1_2_1_ and crystals from the PEG II Suite were of space group P2_1_ (Table 1). Data were collected at the PXII X10SA beamline of the Swiss Light Source (SLS) and was indexed and processed with XDS (Kabsch, 1993). Molecular replacement using different Arl structures was done with MOLREP and PHASER from the CCP4 package (Collaborative Computational Project Number 4, 1994). A model of the BARTL1^133^ sequence generated by the PHYRE threader based on BART (3DOE) was used in molecular replacement to solve the BARTL1^133^ structure in the complex. The structure was refined using REFMAC5 (Murshudov et al., 1997) to the following resolutions (Ramachandran statistics in parentheses): Arl3∙GppNHp∙BARTL1^133^ native P2_1_2_1_2_1_ to 2.2 Å (99.0% favored, 1.0% allowed, 0% outliers) and P2_1_ to 2.0 Å (97.6% favored, 2.4% allowed, 0% outliers). Structures were deposited in the RCSB PDB databank with entry codes PDB: 4ZI2 and 4ZI3, respectively. For data and refinement statistics, see Table 1. All figures were produced using PYMOL (DeLano Scientific).

### Analytical Size-Exclusion Chromatography

Complex formation of Arl3 or Arl3ΔN with BARTL1 or BARTL1^133^ was investigated by analytical size-exclusion chromatography using a Superdex200 10/300 column (GE Healthcare). 0.5 mg of Arl3 protein was incubated with a 10-fold molar excess of GDP or GppNHp for 2 hr at room temperature. The mix was supplemented with 0.5 mg of full-length or truncated BARTL1 or BARTL1^133^, applied to the size-exclusion chromatography column, and eluted with one column volume of buffer M. The elution profile was recorded and eluted fractions analyzed by SDS-PAGE.

### Determination of Dissociation Rates by Stopped Flow

A preformed complex of 2 μM Arl3∙GppNHp with 1 μM FITC-BARTL1^133^ was shot together with a 50-fold excess of unlabeled BARTL1^133^. The dissociation of the complex was followed by monitoring the polarization signal at excitation and emission wavelengths of FITC at 490 and 520 nm, respectively. Single exponential functions were fitted to the data using Grafit5 (Erithacus Software) to obtain the *k*_off_ values.

### Affinity Measurements

Arl3^WT^, Arl3^L4D^, Arl3^F51A^, and Arl2^WT^ were loaded with mant-GDP or mant-GppNHp (Pharma Waldhof) overnight at 12°C by incubation with a 1.5-fold molar excess of nucleotide, and purified the following day on a Desalting Column in buffer M (Veltel et al., 2008b). Nucleotide loading was determined by high-performance liquid chromatography measurements on a C18 column. Polarization data were recorded with a Fluoromax-4 spectrophotometer (Jobin Yvon), with excitation and emission wavelengths of mant-nucleotides at 366 and 450 nm, respectively. Binding affinities of Arl3^WT^, Arl3^L4D^, Arl3^F51A^, and Arl2^WT^ to BARTL1 and BARTL1^133^ were measured by monitoring the polarization signal during titration of 1 μM Arl3 loaded with the respective nucleotide with increasing amounts of the interaction partner at 20°C in buffer M. Cy5-BARTL1^133^ was used to determine binding affinities to Arl3^WT^, Arl3^L4D^, Arl3^F51A^, Arl3ΔN, and Arl2^WT^ bound to GppNHp. 0.2 μM Cy5-BARTL1^133^ was titrated with increasing amounts of Arl proteins, and polarization data were recorded with excitation and emission wavelengths of Cy5 at 650 and 670 nm, respectively. Obtained data points were fitted to a first-order reaction using Grafit5 (Erithacus Software) to obtain the dissociation constant, *K*_D_.

### Generation of Stable Cell Lines

Mouse renal epithelial Flp-In cells from the inner medullary collecting duct (IMCD3 Flp-In; kind gift from M.V. Nachury) were cultured at 37°C and 5% CO_2_ in DMEM/F12, HEPES (Life Technologies) complemented with 10% fetal bovine serum (FBS), and 1% L-glutamine. Stable cell lines were generated as previously described (Sang et al., 2011, Torres et al., 2009). In short, the parental IMCD3 Flp-In cell line contains a stably integrated FRT cassette and was co-transfected with pOG44 coding an FLP recombinase, and the appropriate construct cloned into pgLAP5 vector (Addgene), coding for a C-terminal S- and GFP-tag, using Lipofectamine 2000 (Life Technologies). Selection by supplementing the media with 200 μg/ml hygromycin (Merck) for successful stable genomic integration was carried out, and expression of the GFP fusion protein was checked by western blot using an anti-GFP antibody (1:500; Santa Cruz Biotechnology).

### Knockdown

Stable IMCD3 Flp-In cell lines expressing Arl3 or CCDC104/BARTL1 were plated on poly-L-lysine-coated coverslips. After 24 hr, cells were transfected with 100 nM siRNAs directed against mouse *ARL3* or mouse *CCDC104* and a negative control siRNA, using Lipofectamine 2000 following the manufacturer’s recommendations. FlexiTube siRNA oligos SI00214963 directed against *ARL3*, FlexiTube siRNA oligos SI00848855 directed against *CCDC104*, and negative control siRNA (scrambled) oligo 1027310 were used (Qiagen). 48 hr after transfection of siRNAs against *ARL3*, cells were serum-starved for 24 hr or, 24 hr after transfection of siRNAs against *CCDC104* and direct serum starvation, cells were treated for immunofluorescence microscopy as described below. Images were collected using identical settings for each sample.

### Imaging by Microscopy

IMCD3 stables expressing GFP fusion proteins were plated on poly-L-lysine-coated coverslips and cilia induced by 48 hr of serum starvation. Cells were washed in PBS and fixed with 4% formaldehyde for 20 min (AcTub) or 2% formaldehyde and 50% ice-cold methanol for 15 min at 4°C (γ-Tub). Cells were permeabilized with 0.3% Triton X-100 in cytoskeletal buffer for 10 min. Cells were rinsed in 0.1% Tween 20 in PBS and blocked in 10% FBS in PBS for 30 min. For immunostaining of primary cilia, mouse 611B1 anti-acetylated α-tubulin antibody (1:5000; Sigma-Aldrich) or anti-Arl13B antibody (1:1000; Proteintech); and for basal body staining anti-γ-tubulin antibody (clone GTU-88, 1:1500; Sigma-Aldrich) and Arl3 staining anti-Arl3 antibody (1:500; Novus Biologicals) in 10% FBS in PBS were incubated overnight at 4°C. Alexa Fluor 647 or 405 anti-mouse or Alexa Fluor 647 anti-rabbit antibody (1:800; Life Technologies) was added for 45 min at room temperature. Coverslips were rinsed three times in 0.1% Tween 20 in PBS and once in PBS. Nuclei were stained with DAPI (Serva) diluted 1:10,000 in PBS for 1 min. Coverslips were fixed on glass slides with Mowiol (Merck). Images were taken using an Olympus IX81 microscope with a CCD camera and a 60× NA 1.35 objective. In all cases at least three independent staining experiments were carried out, and 100 cells were used for analysis.

## Author Contributions

Protein preparation, biochemical/biophysical measurements, generation of stable cell lines, crystallization, X-ray data analysis, and manuscript preparation were carried out by M.L. C.K. cloned various constructs and helped in purifying proteins. S.K. carried out the generation of stable cell lines, knockdown experiments, immunostaining, and analysis by fluorescence microscopy. S.M.L. examined triple complex formation by polarization experiments. TAP experiments were carried out with the help of S.E.C.v.B. TAP eluates were analyzed using mass spectrometry by N.H. and K.B., and data were processed by J.v.R. under supervision of M.U. and R.R., respectively. Project design, supervision, and manuscript preparation were supervised by A.W.

## Figures and Tables

**Table 1:** Data Collection and Refinement Statistics from Molecular Replacement

	Arl3∙GppNHp∙CCDC104^133^	Arl3∙GppNHp∙CCDC104^133^
PDB ID	4ZI2	4ZI3

**Data Collection**		

Space group	P 2_1_ 2_1_ 2_1_	P1 2_1_ 1
Cell dimensions		
*a*, *b*, *c* (Å)	69.70, 98.60, 102.43	51.55, 67.72, 98.47
α, β, γ (°)	90.00, 90.00, 90.00	90.00, 102.65, 90.00
Resolution (Å)	29.73–2.20 (2.30–2.20)	28.95–2.00 (2.10–2.00)
*R*_sym_ or *R*_merge_	11.0 (54.0)	5.8 (39.8)
*I*/σ*I*	11.87 (3.66)	15.28 (3.72)
Completeness (%)	99.9 (99.9)	99.2 (99.1)
Redundancy	6.55 (6.80)	3.35 (3.24)

**Refinement**		

Resolution (Å)	2.20	2.00
No. of reflections	36,470	44,692
*R*_work_/*R*_free_	0.2087/0.2660	0.1893/0.2427
No. of atoms		
Total	5,442	5,335
Protein	4,993	4,995
Ligand/ion	66	66
Water	383	274
*B* factors	37.54	45.63
Rmsd		
Bond lengths (Å)	0.008	0.008
Bond angles (°)	1.098	1.145

Values in parentheses are for highest-resolution shell.

## Supplemental Information

Document S1. Figures S1–S6, Table S1, and Supplemental Experimental Procedures

4ZI2 – Arl3∙GppNHp∙BARTL1 complex in P21 21 21

4ZI3 – Arl3∙GppNHp∙BARTL1 complex in P1 21 1

Document S2. Article plus Supplemental Information
