# Supplementary material for: The Interaction of CCDC104/BARTL1 with Arl3 and Implications for Ciliary Function
Source: Structure. 2015 Nov 3;23(11):2122–32. doi: 10.1016/j.str.2015.08.016 (PMC4635315; doi:10.1016/j.str.2015.08.016)
Supplement: Document S1. Figures S1–S6, Table S1, and Supplemental Experimental Procedures [file mmc1.pdf]

Structure, Volume *23*

Supplemental Information

The Interaction of CCDC104/BARTL1 with Arl3 and Implications for Ciliary Function

Mandy Lokaj, Stefanie K. Kösling, Carolin Koerner, Sven M. Lange, Sylvia E.C. van Beersum, Jeroen van Reeuwijk, Ronald Roepman, Nicola Horn, Marius Ueffing, Karsten Boldt, and Alfred Wittinghofer

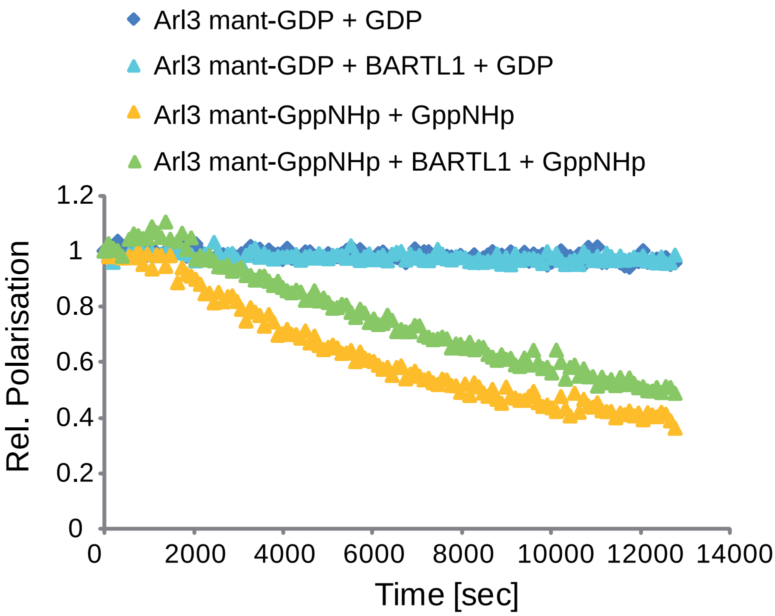

Arl3 mant-GDP + GDP
Arl3 mant-GDP + BARTL1 + GDP
Arl3 mant-GppNHp + GppNHp
Arl3 mant-GppNHp + BARTL1 + GppNHp
Rel. Polarisation
1.2
1
0.8
0.6
0.4
0.2
0
0
2000
4000
6000
8000
10000
12000
14000
Time [sec]

Figure S1

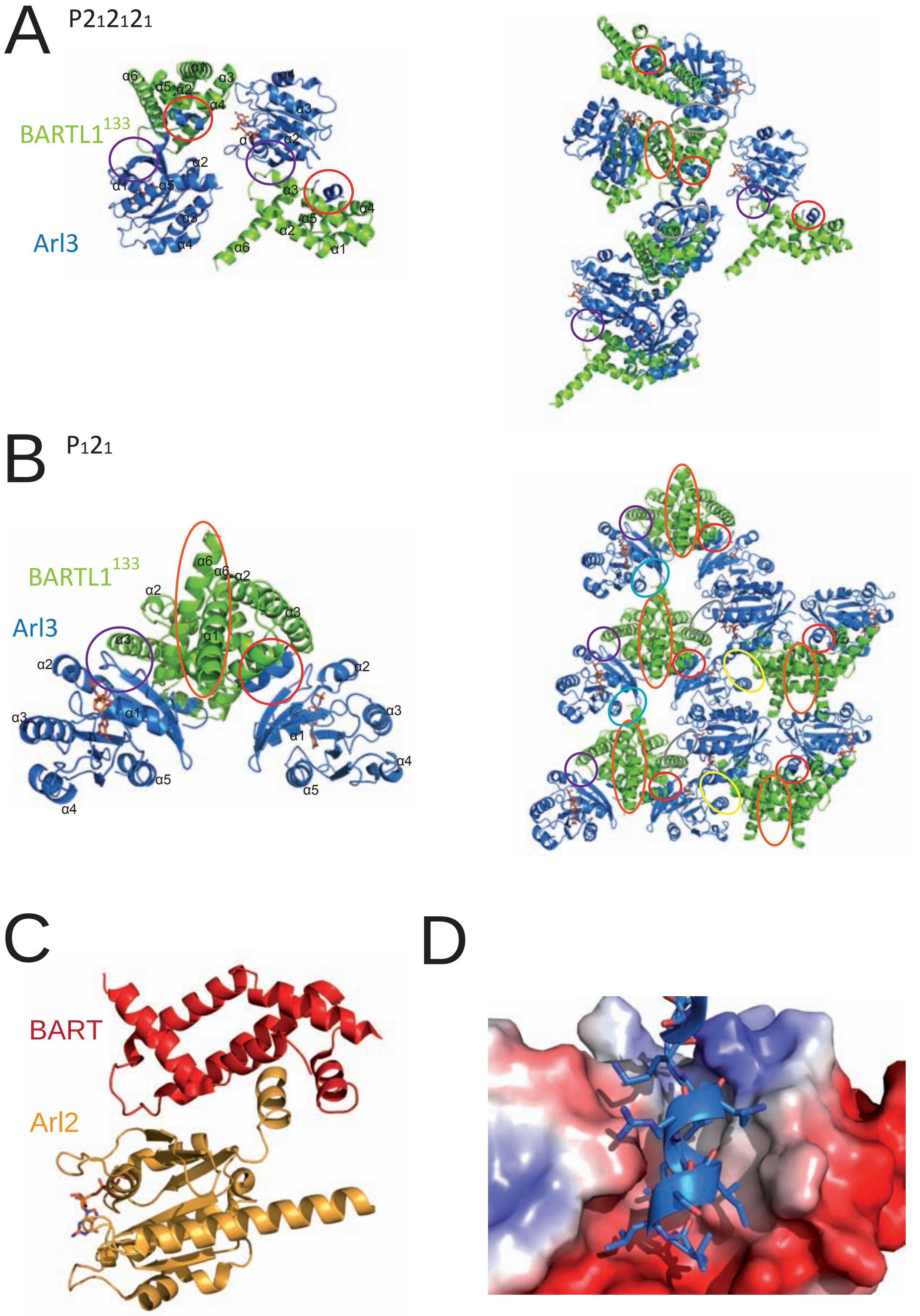

Figure S2

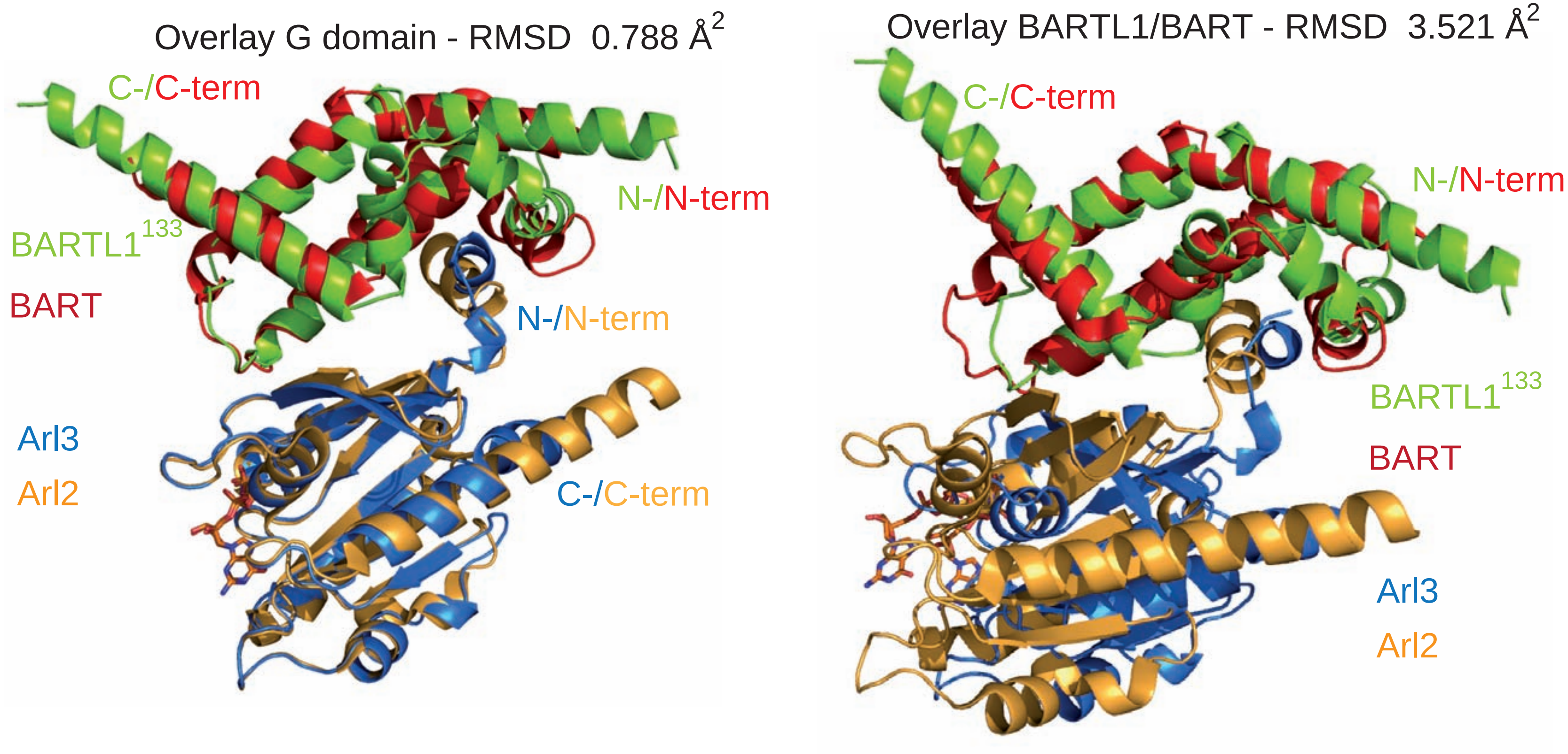
Overlay G domain - RMSD 0.788 Å2
C-/C-term
N-/N-term
BARTL1133
BART
N-/N-term
Arl3
Arl2
C-/C-term
Overlay BARTL1/BART - RMSD 3.521 Å2
C-/C-term
N-/N-term
BARTL1133
BART
Arl3
Arl2

Figure S3

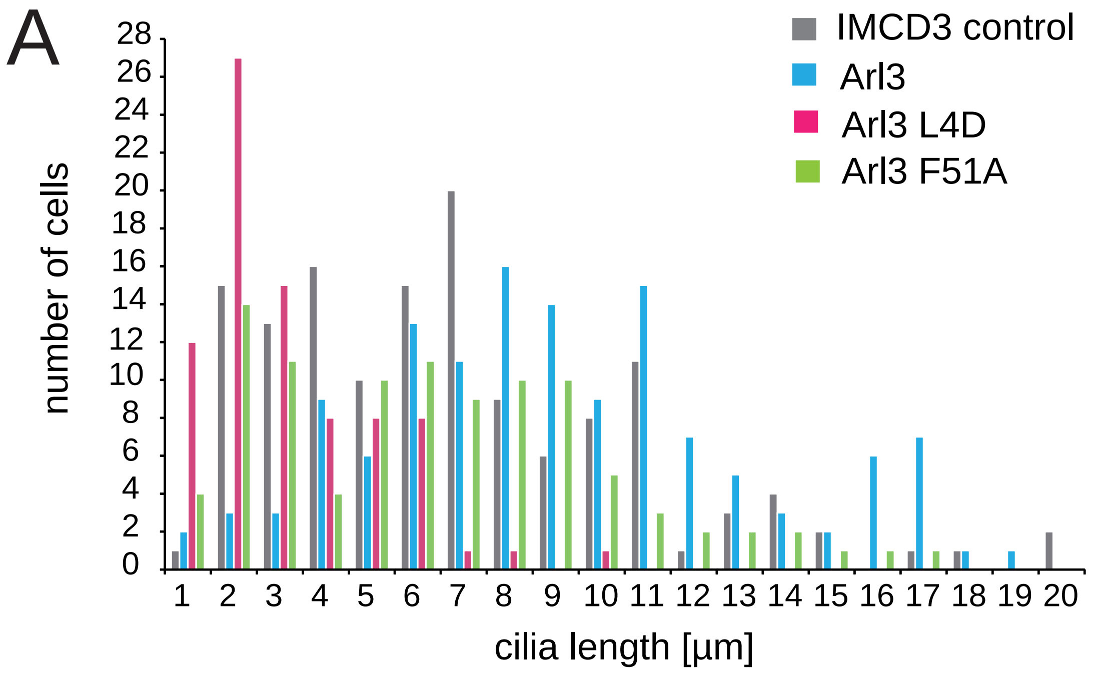

cell line	average cilia length [µm]	% ciliated cells
IMCD3	7	73
Arl3	9	75
Arl3 L4D	3	73
Arl3 F51A	6	76

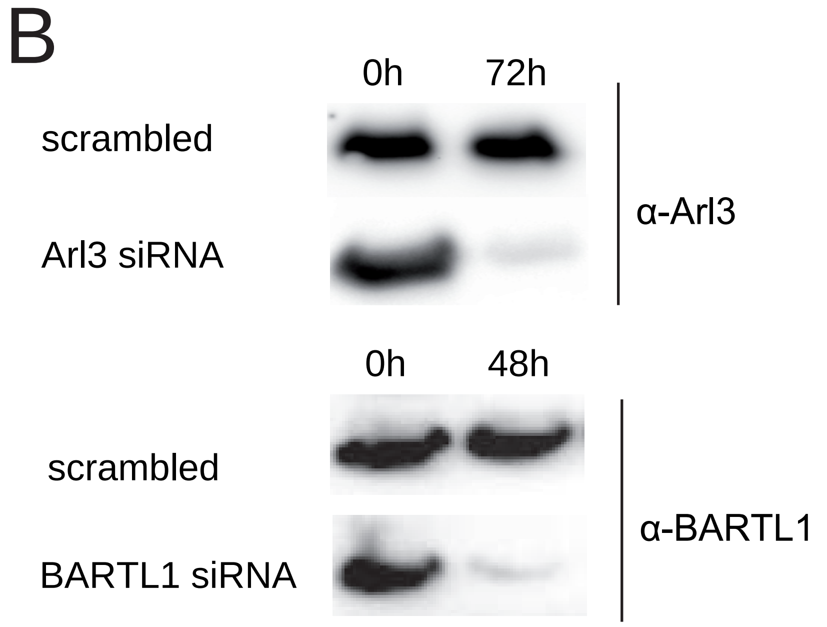

Figure S4

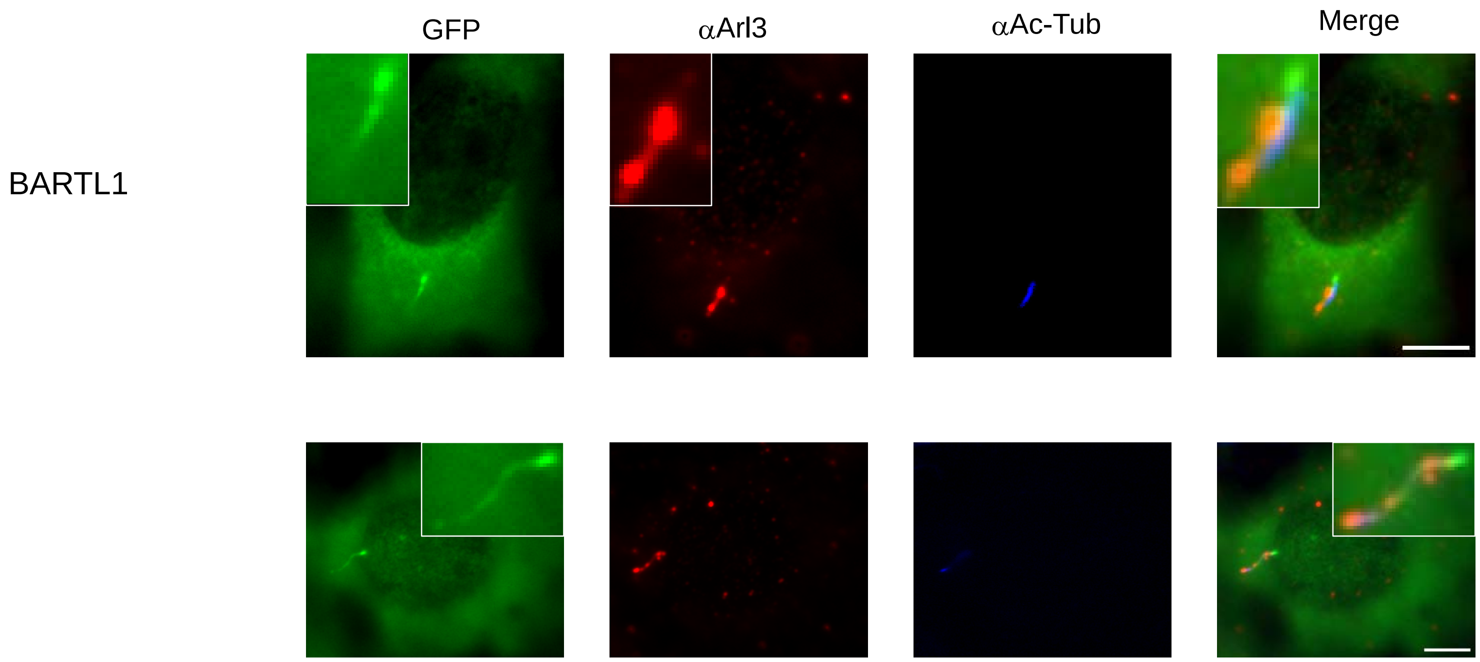

Figure S5

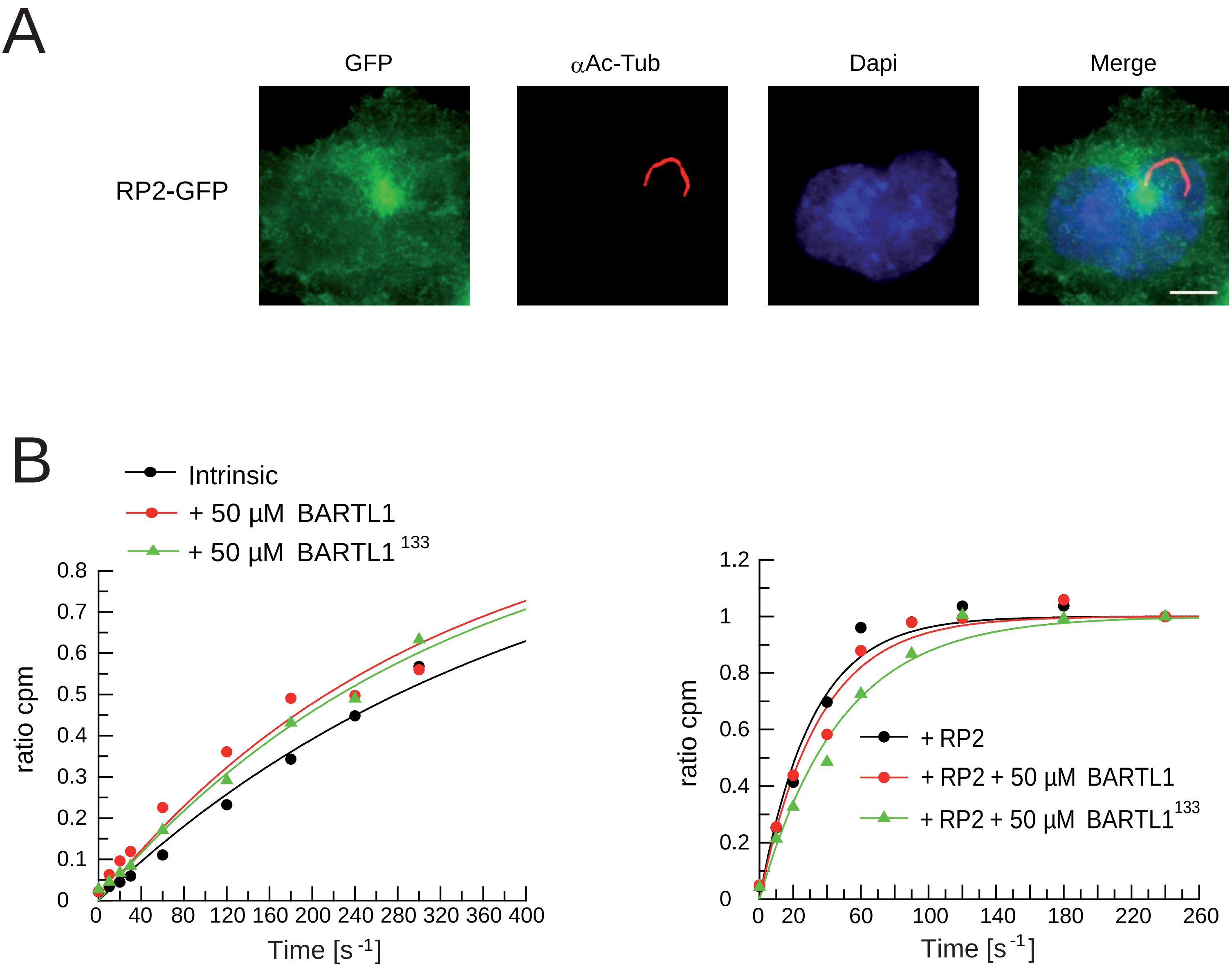

	Rate constants cpm/s^{-1}
Arl3 intrinsic	0.0025 ± 9.16*10^{-5}
+ BARTL1	0.0032 ± 2.00*10^{-4}
+ BARTL1^{133}	0.0031 ± 9.23*10^{-5}

	Rate constants cpm/s^{-1}
Arl3	
+ RP2	0.0325 ± 0.0028
+ RP2 + BARTL1	0.0286 ± 0.0024
+ RP2 + BARTL1^{133}	0.0209 ± 0.0014

Figure S6

Figure S1, Related to Figure 3. CCDC104/BARTL1 is an effector but no GEF for Arl3. Fluorescence polarisation measurements to test for GEF activity of BARTL1. Relative fluorescence polarisation values were plotted against the time. Nucleotide exchange was induced by addition of 100-fold excess of unlabelled GDP or GppNHp, respectively to 1 µM Arl3 bound to either mant-GDP or mant-GppNHp in the presence and absence of BARTL1.

Figure S2, Related to Figure 4. Crystal Contacts. (**A**) The Arl3•GppNHp•BARTL1^{133} complex (pdb: 4ZI2) crystallized in space group $P2_12_12_1$ and the asymmetric unit contained two Arl3 (blue) and two BARTL1^{133} (green) molecules (left panel). Two biological assemblies via interaction Area 1 (red circle) and 2 (lilac circle) (see main text) can be found. Crystal contacts are formed via dimer formation of BARTL1^{133} involving its α2, α5 and α6 helices (orange circle). The α5 helix of Arl3 is forming further crystal contacts to the α2 and α6 helices of a neighbouring BARTL1^{133} molecule (grey circle). (**B**) The Arl3•GppNHp•BARTL1^{133} complex (pdb: 4ZI3) crystallized in space group P_12_1 and the asymmetric unit contained two Arl3 (blue) and two BARTL1^{133} (green) molecules (left panel). Within the asymmetric unit BARTL1^{133} is forming a dimer involving its α2, α5 and α6 helices (orange circle) and each BARTL1^{133} is contacting Arl3 via interaction Area 1 (red circle) and 2 (lilac circle) (see main text). Crystal contacts are formed by the α6 helices of both BARTL1^{133} molecules contacting α3 and the loop between β2-β3 of Arl3 (cyan circle). The α3 helix of BARTL1^{133} contacts the loop between α4-β4 of a further Arl3 molecule (grey circle). Additional, the α2 and α3 helices of Arl3 are contacting α3 and α1 of a neighbouring BARTL1^{133} molecule (yellow circle). **(C)** The complex of Arl2•GTP•BART (pdb: 3DOE) crystallized in space group $P2_1$ (Zhang et al., 2009). The asymmetric unit contained only one Arl2 (orange) and one BART (red) molecule representing the biological assembly. (**D**) Surface representation of BARTL1 (red – acidic, blue – basic, white – hydrophobic patches) showing the groove in which N-term of Arl3 (blue) is buried.

Figure S3, Related to Figure 5. Overlay of Arl2•BART (pdb: 3DOE) and Arl3•BARTL1 (pdb: 4ZI2). Superimposition of the G domain of Arl2 (orange) and Arl3 (blue) (left panel) and BART (red) and BARTL1 (green) (right panel) of both structures. N- and C-termini of proteins and rmsd values are indicated.

Figure S4, Related to Figure 7. Further analysis of stable cell lines. (**A**) Quantification of cilia number and length for IMCD3 control cells and cells stably expressing $Arl3^{WT}$; $Arl3^{L4D}$ and $Arl3^{F51A}$. The cilia length of 100 cells was plotted according to the determined cilia length. (**B**) Samples of siRNA treated stable cell lines used for imaging (see Figure 7) compared to cells treated with scrambled control siRNA were subjected to SDS-PAGE and analysed by Western Blot: anti-Arl3 antibody (1:500; Novus Biologicals) and anti-CCDC104/BARTL1 antibody (1:500, Abnova).

Figure S5, Related to Figure 2. Co-Staining of Arl3 and BARTL1. Two representative IMCD3 cells stably expressing BARTL1-GFP in which endogenous Arl3 and acetylated α-tubulin were stained following serum starvation and fixation. White bar indicates 5 µm.

Figure S6, Related to Figure 8. GTP hydrolysis measurement. (**A**) IMCD3 cells stably expressing, C-terminally tagged full-length human RP2-GFP were and immunostained for acetylated α-tubulin (AcTub) and the nucleus (DAPI). White bar indicates 5 µm. (**B**) Intrinsic (left panel) or RP2 stimulated (right panel) GTP hydrolysis of either 10 µM Arl3 loaded with 60 nM ^{32}P-GTP/10 µM GTP alone or in presence of 0.1 µM RP2 and/or 50 µM BARTL1 or $BARTL1^{133}$. Rough observed rate constants (and standard deviations) are indicated below in the table.

Proteins identified by mass spectrometry from tandem affinity proteomics experiments in HEK293T cells			Arl3_D129N_C-TAP_Exp1		Arl3_D129N_C-TAP_Exp2	
EntrezGeneSymbol	EntrezGeneFullName	SwissProt_2013_02_Accession	unique peptides	sequence coverage	unique peptides	sequence coverage
ABHD10	abhydrolase domain containing 10	Q9NUJ1	4	0,19	4	0,16
AIFM1	apoptosis-inducing factor, mitochondrion-associated, 1	O95831	15	0,36	24	0,48
ARL2BP	ADP-ribosylation factor-like 2 binding protein	Q9Y2Y0	5	0,39	6	0,37
ARL3	ADP-ribosylation factor-like 3	P36405	12	0,68	10	0,67
C20orf194	chromosome 20 open reading frame 194	Q5TEA3	34	0,41	35	0,43
CFAP36	cilia and flagella associated protein 36	Q96G28	3	0,13	9	0,37
PDE6D	phosphodiesterase 6D, cGMP-specific, rod, delta	O43924	5	0,35	5	0,35
RPL23	ribosomal protein L23	P62829	2	0,11	2	0,16
UBB	ubiquitin B	P0CG47	4	0,17		
UNC119	unc-119 homolog (C. elegans)	Q13432	7	0,44	8	0,53
UNC119B	unc-119 homolog B (C. elegans)	A6NIH7	17	0,84	17	0,82
YWHAB	tyrosine 3-monooxygenase/tryptophan 5-monooxygenase activation proteir	P31946	5	0,45	5	0,58
YWHAG	tyrosine 3-monooxygenase/tryptophan 5-monooxygenase activation proteir	P61981	4	0,32	6	0,49
YWHAH	tyrosine 3-monooxygenase/tryptophan 5-monooxygenase activation proteir	Q04917	2	0,18	4	0,35
YWHAQ	tyrosine 3-monooxygenase/tryptophan 5-monooxygenase activation proteir	P27348	7	0,40	11	0,56
YWHAZ	tyrosine 3-monooxygenase/tryptophan 5-monooxygenase activation proteir	P63104	7	0,47	14	0,64

Table S1. SF-TAP analysis with over-expressed C-terminally SF-TAP-tagged Arl3_D129N in HEK293T cells. Shown are the number of unique identified peptides as well as the sequence coverage for each protein detected by mass spectrometry in 2 experiments. Proteins identified in the SF-TAP analysis of empty vector control experiments were removed.

Exp1

Q96G28 (100%), 39.447,1 Da

Coiled-coil domain-containing protein 104 OS=Homo sapiens GN=CCDC104 PE=1 SV=2

3 exclusive unique peptides, 3 exclusive unique spectra, 3 total spectra, 46/342 amino acids (13% coverage)

MAAEEEDEVE WVVESIAGFL R**GPDWSIPIL** **DFVEQK**CEVF DDEEESKLTY TEIHQEYKEL VEKLLEGYLK EIGINEDQFQ
EACTSPLAK**T** **HTSQAILQPV** **LAAEDFTIFK** AMMVQK**NIEM** **QLQAIR**IIQE RNGVLPDCLT DGSDVVSDLE HEEMKILREV
LRKSKEEYDQ EEERKRKKQL SEAKTEEPTV HSSEAAIMNN SQGDGEHFAH PPSEVKMHFA NQSIEPLGRK VERSETSSLP
QKDLKIPGLE HASIEGPIAN LSVLGTEELR QREHYLKQKR DKLMSMRKDM RTKQIQNMEQ KGKPTGEVEE MTEKPEMTAE
EKQTLLKRRL LAEKLKEEVI NK

Exp2

Q96G28 (100%), 39.447,1 Da

Coiled-coil domain-containing protein 104 OS=Homo sapiens GN=CCDC104 PE=1 SV=2

9 exclusive unique peptides, 9 exclusive unique spectra, 9 total spectra, 128/342 amino acids (37% coverage)

MAAEEEDEVE WVVESIAGFL RGPDWSIPIL DFVEQKCEVF DDEEESKLTY TEIHQEYKEL VEK**LLEGYLK** **EIGINEDQFQ**
EACTSPLAKT **HTSQAILQPV** **LAAEDFTIFK** AMMVQK**NIEM** **QLQAIRIIQE** **RNGVLPDCLT** **DGSDVVSDLE** **HEEMK**ILREV
LRKSKEEYDQ EEERKRKKQL SEAKTEEPTV HSSEAAIMNN SQGDGEHFAH PPSEVKMHFA NQSIEPLGRK VER**SETSSLP**
QKDLK**IPGLE** **HASIEGPIAN** **LSVLGTEELR** QREHYLKQKR DKLMSMRKDM RTKQIQNMEQ KGKPTGEVEE MTEKPEMTAE
EKQTLLKRRL LAEK**LKEEVI** **NK**

Supplementary Experimental Procedures

Plasmids and protein purification BARTL1 was amplified by PCR from a cDNA library from a mouse spleen cDNA and a human W38 cDNA library. In this work human full length BARTL1 (UNP:Q96G28) and a shortened mouse BARTL1 (UNP:Q8C6E0) comprising amino acids 1 to 133 were used. Full length BARTL1 was cloned into pProExHTa containing an N-terminal His tag and $BARTL1^{133}$ into pGexET (derivative of pGex4T-1) containing an N-terminal Glutathione-S-transferase fusion followed by a thrombin, TEV and precission cleavage site (order as mentioned). Arl3 (UNP:Q9WUL7) and Arl2 (UNP:Q9D0J4) full length in pET20 as well as Arl3ΔN and Arl2ΔN in pGex4T-1 (Veltel et al., 2008b) were already available. Respective BARTL1 mutants and Arl mutants were generated by mutagenesis PCR. All proteins were expressed in BL21 DE3 codon plus RIL cells at 25°C following induction with 100 µM IPTG at 18°C overnight. Purification was done using GSH-sepharose columns (Amersham/GE Healthcare) which were washed with Wash-Buffer (75 mM Hepes pH 7.5, 300 mM KCl, 5mM $MgCl_2$, 3 mM β-mercaptoethanol and 10 % glycerol). The GST-fusion proteins were eluted with Elution-Buffer (Wash Buffer + 20 mM reduced glutathione). Following cleavage with precission protease overnight residual GST was removed by size exclusion chromatography using a Superdex 200 16/60 (Amersham/GE Healthcare). Arl3 and Arl2 proteins and mutants containing a C-terminal His-tag were purified as previously described (Veltel et al., 2008b). The proteins were stored in buffer M containing 25 mM Hepes pH 7.5, 150 mM KCl, 5 mM $MgCl_2$, 1 mM DTE and 5 % glycerol. The nucleotide content of all G proteins was determined by HPLC measurements. All proteins used displayed full nucleotide loading. Plasmids used for the generation of stable cell lines can be found below in the respective section.

Cy5, FITC Labelling of BARTL1 For $BARTL1^{133}$ the mutant C83A/E59C was constructed for labelling. 1 mg of protein was exchanged into 1 x PBS, 1 mM TCEP and incubated with a

50-fold molar excess of Cy5 or FITC in DMSO, respectively for 3 hours at room temperature and further incubation overnight at 4°C. Following day the excess label was removed by a Desalting Column. The ratio of protein:label was determined 1:3, i.e. 30 % efficiency.

Liposome Sedimentation Assay The phospholipids 1,2-dioleoyl-sn-glycero-3-phosphocholine (DOPC), 1,2-dioleoyl-sn-glycero-3-phospho-(1'-rac-glycerol) sodium salt (DOPG), 1,2-dipalmitoyl-snglycero-3-phospho-(1'-rac-glycerol) sodium salt (DPPG), and 1,2-dipalmitoyl-sn-glycero-3- phosphocholine (DPPC) were purchased from Avanti Polar Lipids (Alabaster, AL). Cholesterol (Chol) was from Sigma-Aldrich.

DOPC:DOPG:DPPC:DPPG:Cholesterol were mixed in a molar ratio of 4:25:5:50:25 and vacuum dried. The dried lipid mix was resuspended in a buffer containing 20 mM Tris pH7.5, 20 mM NaCl, 5 mM MgCl2, 1 mM DTE (buffer L) to a final concentration of 2.8 mM and sonicated at 65 °C for 15 min and subsequently subjected to nine freeze-thaw-vortex cycles. Afterwards, unilamellar vesicles of homogeneous sizes were obtained by using an extruder (Avanti Polar Lipids, Alabaster, AL) with polycarbonate membranes of 200 nm pore size at 65 °C in presence of 40 µM GDP or GppNHp in buffer L. 2.8 mM of 200 µM liposomes were incubated with 20 µM Arl3 bound to GDP or GppNHp, respectively in the presence of 40 µM BARTL1[133] for 30 min at room temperature. Liposomes were pelleted at 125,000 × *g* for 1 h 30 min at 10°C in a TLA-45 rotor. The pellets were resuspended in buffer L, up to the same volume as the supernatant. Equal amounts of the supernatants and resuspended pellets volumes were analyzed by SDS-PAGE.

Measurement of GTP hydrolysis by [γ-32P]GTP charcoal method This was performed as described (Brinkmann et al., 2002; Miertzschke et al., 2011). Briefly, a mix of 10 µM GTP and 60 nM [γ-^{32}P]GTP in Buffer M was supplemented with 10 µM Arl3 bound to GppCH$_2$p to start the intrinsic GTPase reaction at 25°C. For investigation of RP2-stimulated GTP-

hydrolysis, 0.1 µM RP2 was added to start the reaction. The intrinsic and RP2 stimlated GTP-hydrolysis was measured in absence and presence of 50 µM BARTL1 or BARTL1^{133}. Aliquots of 10 µl were taken at certain time points and mixed with 400 µl of charcoal solution (50 g*l-1 charcoal in 20 mM phosphoric acid) to stop the reaction. The charcoal was pelleted and the amount of free ^{32}Pi in the supernatant determined by scintillation counting. Data was plotted by showing the ratio of specific counts of supernatant over total counts of sample at each point. Data points were fitted to a first-order reaction to obtain rough kobs.

Tandem affinity purification. HEK293T (human embryonic kidney, ATCC) cells were transfected for 48 hours with SF-TAP-Arl3^{D129N} using polyethyleneimine (PEI, Polysciences) as a transfection reagent. Following transfection, cells were lysed in lysis buffer containing 30 mM Tris–HCl (pH 7.4), 150 mM NaCl, 0.5% Nonidet-P40 (NP40), freshly supplemented with protease inhibitor cocktail (Roche), phosphatase inhibitor cocktail II and III (Sigma), for 20 minutes at 4°C. The Streptavidin- and FLAG-based tandem affinity purification steps were performed as previously described (Boldt et al., 2009; Gloeckner et al., 2007). 5% of the final eluate was evaluated by SDS-PAGE followed by silver staining, according to standard protocols, while the remaining 95% were subjected to protein precipitation with chloroform and methanol. Protein precipitates were subsequently subjected to mass spectrometry analysis and peptide identification as previously described (Texier et al., 2014). For one step Strep purifications, SF-TAP-tagged proteins and associated protein complexes were purified essentially as described earlier (Gloeckner et al., 2009a). HEK293T cells, transiently expressing the SF-TAP-tagged constructs were lysed in lysis buffer, containing 0.5% Nonidet-P40, protease inhibitor cocktail (Roche) and phosphatase inhibitor cocktails II and III (Sigma-Aldrich) in TBS (30 mM Tris-HCl (pH 7.4), 150 mM NaCl), for 20 minutes at 4°C. After sedimentation of nuclei at 10,000 x g for 10 minutes, the protein concentration was determined by a Bradford assay, before equal amounts of each lysate were transferred to

Strep-Tactin-Superflow beads (IBA) and were incubated for one hour at 4°C on an end-over-end shaker. Then, the resin was washed three times with wash buffer (TBS containing 0.1% NP-40, phosphatase inhibitor cocktail II and III). The protein complexes were eluted by incubation for 10 minutes in Strep-elution buffer (IBA). The eluted samples were concentrated using 10 kDa cut-off VivaSpin 500 centrifugal devices (Sartorius Stedim Biotech) and pre-fractionated using SDS-Page. Afterwards, the samples were subjected to in-gel tryptic cleavage as described elsewhere (Gloeckner et al., 2009b).

Mass spectrometry and data analysis. LC-MS/MS analysis was performed on an Ultimate3000 nano RSLC system (Thermo Scientific) coupled to a LTQ Orbitrap Velos mass spectrometer (Thermo Scientific) by a nano spray ion source. Tryptic peptide mixtures were automatically injected and loaded at a flow rate of 6 µl/min in 0.1% trifluoroacetic acid in HPLC-grade water onto a nano trap column (75 µm i.d. × 2 cm, packed with Acclaim PepMap100 C18, 3 µm, 100 Å; Thermo Scientific). After 5 minutes, peptides were eluted and separated on the analytical column (75 µm i.d. × 25 cm, Acclaim PepMap RSLC C18, 2µm, 100 Å; Thermo Scientific) by a linear gradient from 2% to 35% of buffer B (80% actetonitrile and 0.08% formic acid in HPLC-grade water) in buffer A (2% acetonitrile and 0.1% formic acid in HPLC-grade water) at a flow rate of 300 nl/min over 80 minutes. Remaining peptides were eluted by a short gradient from 35% to 95% buffer B in 5 minutes. The eluted peptides were analyzed by a LTQ Orbitrap Velos mass spectrometer. From the high resolution MS pre-scan with a mass range of 300 to 1500, the ten most intense peptide ions were selected for fragment analysis in the linear ion trap if they exceeded an intensity of at least 200 counts and if they were at least doubly charged. The normalized collision energy for CID was set to a value of 35 and the resulting fragments were detected with normal resolution in the linear ion trap. The lock mass option was activated; the background signal with a mass of 445.12003

was used as lock mass. Every ion selected for fragmentation, was excluded for 20 seconds by dynamic exclusion.

MS/MS data were analyzed, using Mascot (version 2.4.1, Matrix Science, Boston, MA, USA). Mascot was set up to search the human subset of the Swiss Prot database (Release 2013_12, 20248 entries), assuming trypsin as the digestion enzyme. Mascot was searched with a fragment ion mass tolerance of 1 Da and a parent ion tolerance of 10.0 PPM. Oxidation of methionine and was specified as variable modification, iodoacetamide derivative of cysteine as fixed. The Mascot results were loaded in Scaffold (version Scaffold_4.4.1.1, Proteome Software Inc., Portland, OR) to validate MS/MS based peptide and protein identifications. Peptide identifications were accepted if they could be established at greater than 95.0% probability as specified by the Peptide Prophet algorithm (Keller et al., 2002). Protein identifications were accepted if they could be established at greater than 95.0% probability and contained at least 2 identified peptides. Protein probabilities were assigned by the Protein Prophet algorithm (Nesvizhskii et al., 2003). Proteins, which contained similar peptides and could not be differentiated based on MS/MS analysis alone, were grouped to satisfy the principles of parsimony.

Quantification of cilia number and length

Cilia length quantification of parental IMCD3 FlpIn cells and stable Arl3 WT, Arl3 L4D, Arl3 F51A cell lines was performed using Fiji software. After setting the scale, the length was measured by hand using the segmented line tool. For each cell line, approximately 100 cells were analyzed. Data were illustrated in Microsoft Excel plotting rounded cilia length values (µm) against the number of corresponding cells and average length values were calculated for each cell line.

References

Boldt, K., van Reeuwijk, J., Gloeckner, C.J., Ueffing, M., and Roepman, R. (2009). Tandem affinity purification of ciliopathy-associated protein complexes. Methods Cell Biol. *91*, 143–160.

Brinkmann, T., Daumke, O., Herbrand, U., Kühlmann, D., Stege, P., Ahmadian, M.R., and Wittinghofer, A. (2002). Rap-specific GTPase activating protein follows an alternative mechanism. J Biol Chem *277*, 12525–12531.

Gloeckner, C.J., Boldt, K., Schumacher, A., Roepman, R., and Ueffing, M. (2007). A novel tandem affinity purification strategy for the efficient isolation and characterisation of native protein complexes. Proteomics *7*, 4228–4234.

Gloeckner, C.J., Boldt, K., and Ueffing, M. (2009a). Strep/FLAG tandem affinity purification (SF-TAP) to study protein interactions. Curr. Protoc. Protein Sci. *Chapter 19*, Unit19.20.

Gloeckner, C.J., Boldt, K., Schumacher, A., and Ueffing, M. (2009b). Tandem affinity purification of protein complexes from mammalian cells by the Strep/FLAG (SF)-TAP tag. Methods Mol. Biol. *564*, 359–372.

Keller, A., Nesvizhskii, A.I., Kolker, E., and Aebersold, R. (2002). Empirical statistical model to estimate the accuracy of peptide identifications made by MS/MS and database search. Anal. Chem. *74*, 5383–5392.

Miertzschke, M., Koerner, C., Vetter, I.R., Keilberg, D., Hot, E., Leonardy, S., Søgaard-Andersen, L., and Wittinghofer, A. (2011). Structural analysis of the Ras-like G protein MglA and its cognate GAP MglB and implications for bacterial polarity. EMBO J. *30*, 4185–4197.

Nesvizhskii, A.I., Keller, A., Kolker, E., and Aebersold, R. (2003). A statistical model for identifying proteins by tandem mass spectrometry. Anal. Chem. *75*, 4646–4658.

Texier, Y., Toedt, G., Gorza, M., Mans, D.A., van Reeuwijk, J., Horn, N., Willer, J., Katsanis, N., Roepman, R., Gibson, T.J., et al. (2014). Elution profile analysis of SDS-induced subcomplexes by quantitative mass spectrometry. Mol. Cell. Proteomics *13*, 1382–1391.

Zhang, T., Li, S., Zhang, Y., Zhong, C., Lai, Z., and Ding, J. (2009). Article Crystal Structure of the ARL2-GTP-BART Complex Reveals a Novel Recognition and Binding Mode of Small GTPase with Effector. Struct. Des. *17*, 602–610.
